# PINK1 is a target of T cell responses in Parkinson’s disease

**DOI:** 10.1172/JCI180478

**Published:** 2024-12-17

**Authors:** Gregory P. Williams, Antoine Freuchet, Tanner Michaelis, April Frazier, Ngan K. Tran, João Rodrigues Lima-Junior, Elizabeth J. Phillips, Simon A. Mallal, Irene Litvan, Jennifer G. Goldman, Roy N. Alcalay, John Sidney, David Sulzer, Alessandro Sette, Cecilia S. Lindestam Arlehamn

**Affiliations:** 1 Center for Autoimmunity and Inflammation, La Jolla Institute for Immunology, La Jolla, California, USA.; 2 Aligning Science Across Parkinson’s (ASAP) Collaborative Research Network, Chevy Chase, Maryland, USA.; 3 Department of Neurology, Columbia University, Division of Molecular Therapeutics, New York State Psychiatric Institute, New York, New York, USA.; 4 Institute for Immunology and Infectious Diseases, Murdoch University, Perth, Western Australia, Australia.; 5 Vanderbilt University School of Medicine, Nashville, Tennessee, USA.; 6 Department of Neuroscience, UCSD, La Jolla, California, USA.; 7 JPG Enterprises LLC; prior: Shirley Ryan AbilityLab and Northwestern University Feinberg School of Medicine, Chicago, Illinois, USA.; 8 Department of Neurology, Columbia University Irving Medical Center, New York, New York, USA.; 9 Tel Aviv Sourasky Medical Center, Tel Aviv, Israel.; 10 Departments of Psychiatry and Pharmacology, Columbia University, New York State Psychiatric Institute, New York, New York, USA.; 11 Department of Medicine, UCSD, La Jolla, California, USA.; 12 Center for Vaccine Research, Department of Infectious Disease Immunology, Statens Serum Institut, Copenhagen, Denmark.

**Keywords:** Autoimmunity, Immunology, Adaptive immunity, Parkinson disease, T cells

## Abstract

Parkinson’s disease (PD) is the second most prevalent neurodegenerative disorder. While there is no curative treatment, the immune system’s involvement with autoimmune T cells that recognize the protein α-synuclein (α-syn) in a subset of individuals suggests new areas for therapeutic strategies. As not all patients with PD have T cells specific for α-syn, we explored additional autoantigenic targets of T cells in PD. We generated 15-mer peptides spanning several PD-related proteins implicated in PD pathology, including glucosylceramidase β 1 (GBA), superoxide dismutase 1 (SOD1), PTEN induced kinase 1 (PINK1), Parkin RBR E3 ubiquitin protein ligase (parkin), oxoglutarate dehydrogenase (OGDH), and leucine rich repeat kinase 2 (LRRK2). Cytokine production (IFN-γ, IL-5, IL-10) against these proteins was measured using a fluorospot assay and PBMCs from patients with PD and age-matched healthy controls. We identified PINK1, a regulator of mitochondrial stability, as an autoantigen targeted by T cells, as well as its unique epitopes, and their HLA restriction. The PINK1-specific T cell reactivity revealed sex-based differences, as it was predominantly found in male patients with PD, which may contribute to the heterogeneity of PD. Identifying and characterizing PINK1 and other autoinflammatory targets may lead to antigen-specific diagnostics, progression markers, and/or novel therapeutic strategies for PD.

## Abstract 2

PINK1, a mitochondrial-associated protein, has been identified as an autoantigen targeted by T cells from patients with Parkinson’s disease. This reactivity was predominantly found in male patients with Parkinson’s Disease, which may contribute to the heterogeneity of PD.

## Graphical abstract

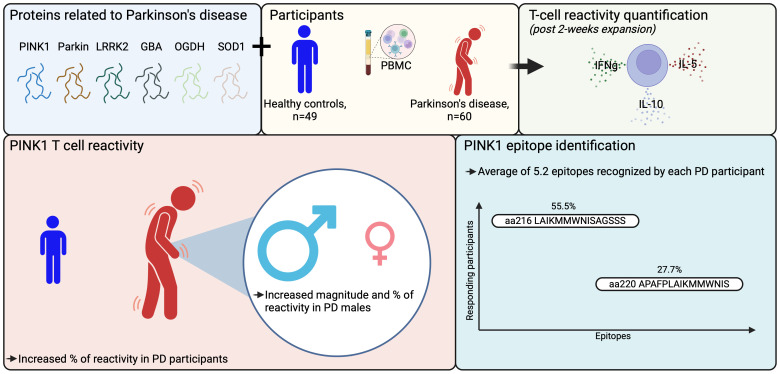

## Introduction

Parkinson’s disease (PD) pathobiology is characterized by the formation of aggregated α-synuclein (α-syn) and subsequent neurodegeneration (1). This prominent role of α-syn in PD development is supported by reports that the gain-of-function genetic variation in the *SNCA* gene (coding for α-syn) can be causal in rare inherited forms of PD (2) or increase one’s risk of developing idiopathic PD (3). However, several other genes and their proteins are associated with the pathology and development of PD. For example, perturbations in PTEN induced kinase 1 (PINK1) (4), Parkin RBR E3 ubiquitin protein ligase (parkin) (5), glucosylceramidase β 1 (GBA) (6), and leucine rich repeat kinase 2 (LRRK2) (7) have each been discovered to be major genetic risk factors for the development of PD. Multiple proposed mechanisms surrounding their pathogenicity role include dysfunctional autophagy (8) and/or mitophagy (9).

The identification of changes in the immune system and neuroinflammation (10) observed in PD patients has paved the way for new avenues of research and potential therapies. Within the brain, activation of resident microglia and astrocytes leads to the expression of proinflammatory cytokines, directly inducing neurotoxicity and recruiting immune cells, such as T cells, from the periphery, (11). Initial studies provided evidence that infiltrating CD4^+^ T cells could be found in postmortem PD brain parenchyma (12, 13), but the role of these cells was unclear. In the periphery, our group has shown that some PD patients harbor CD4^+^ and CD8^+^ T cells recognizing α-syn (14–16). Furthermore, another group recently showed that these synuclein-specific T cells are associated with neurodegenerative signaling in PD and Lewy body dementia, a related synucleinopathy (17). α-Syn–specific T cells may thus mediate neuronal damage (18). The presence of the α-syn–specific T cells correlates with PD duration (15, 16). Other groups have identified a role for CD8^+^ T cells in PD as well as, for example, changes in specific subpopulations (19, 20). As the prodromal phase can start as early as 20 years before diagnosis, early diagnosis and/or monitoring tools can be developed (21).

Recently, PINK1, parkin, GBA, and LRRK2 proteins have been linked to the activation of the immune system (21–23). Additionally, superoxide dismutase 1 (SOD1) has been proposed to be implicated in neurodegeneration (24–26) and oxoglutarate dehydrogenase (OGDH) is involved in preclinical disease models (22), suggesting they could be targeted by T cells. Thus, given that not all patients with PD have α-syn–specific T cells, even at early time points following diagnosis, we hypothesize that additional autoantigens may exist. There are also known phenotypic and biological differences between men and women who have PD (27), and so we sought to further investigate sex-based differences.

To address our hypothesis, we screened a cohort of individuals with PD and age-matched healthy controls (HCs) using peptide pools targeting several PD-associated proteins (PINK1, LRRK2, parkin, GBA, SOD1, and OGDH) and measured the resulting T cell responses. Here, we report that PD patients have more frequent responses with a higher magnitude of cytokine production toward the mitochondrial-associated protein PINK1. This difference was driven by male PD patients, providing a sex-specific difference in antigen recognition. We further report the identification of specific PINK1 epitopes mediating the autoantigenic T cell response. These findings indicate additional antigenic targets in PD and emphasize the promise of potential immune-based biomarkers and therapies in its treatment.

## Results

### Screening PD-related proteins for autoantigenic T cell responses.

**Figure 1:**
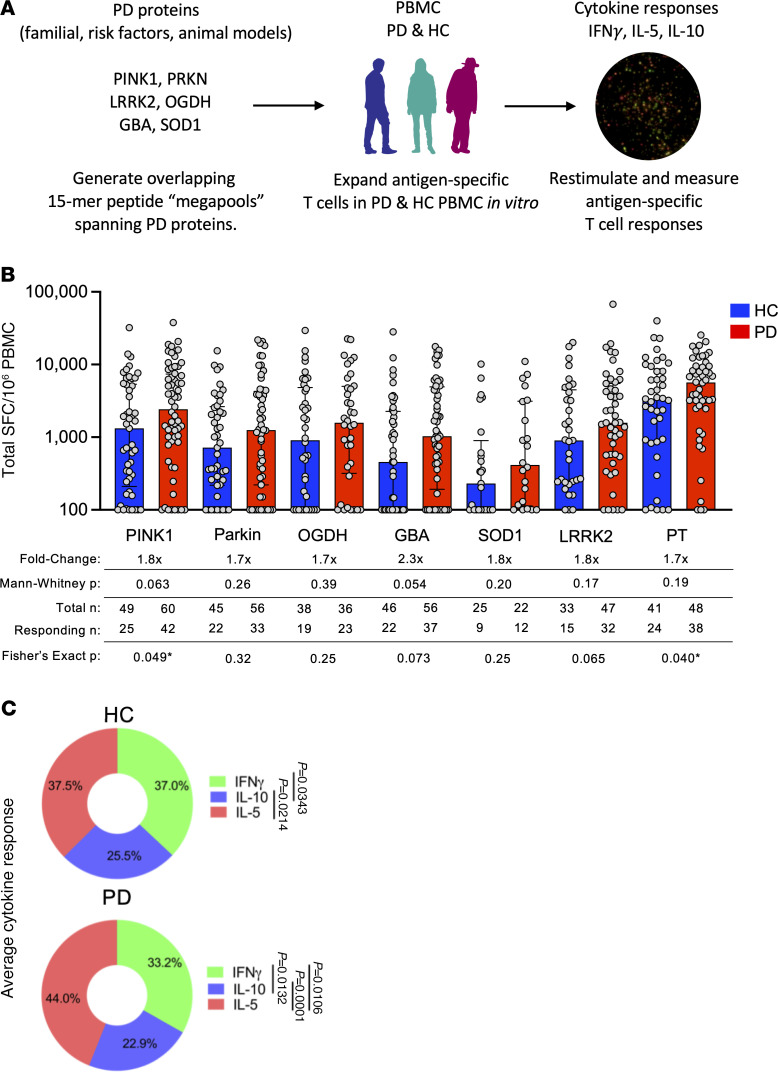
Screening PD-related proteins for autoantigenic T cell responses. (**A**) Experimental design for the screening of PD-related proteins. (I) 15-mer peptides spanning PD-related proteins: PINK1 (117 peptides), PARKIN (94 peptides), OGDH (203 peptides), GBA (106 peptides), SOD1 (34 peptides), LRRK2 (80 predicted peptides), and PT as a control (132 peptides). (II) Peptide pools were incubated at a concentration of 5 ug/mL with PBMCs from PD participants and age-matched HCs for 14 days. (III) Restimulation of cultured PBMCs with the initial antigen pools and subsequent determination of antigen-specific cytokine production using Fluorospot. DMSO and PHA stimuli were used as negative and positive controls, respectively, for each participant/pool combination. (**B**) Magnitude of total cytokine response (sum of IFN-γ, IL-5, and IL-10) to neuroantigens and control PT between HCs (blue bars) and PD (red bars), each circle representing an individual participant. Median ± interquartile range displayed. Fold-change is in comparison to HC response. Two-tailed Mann-Whitney tests were performed between HC and PD antigen-cytokine values. Two-tailed Fisher’s exact tests were performed using the geometric mean of the HC group for each individual antigen as a cutoff for the test. PINK1 (PD, *n* = 39; HC, *n* = 39), PARKIN (PD, *n* = 37; HC, *n* = 39), OGDH (PD, *n* = 36; HC, *n* = 38), GBA (PD, *n* = 37; HC, *n* = 36), SOD1 (PD, *n* = 24; HC, *n* = 25), LRRK2 (PD, *n* = 26; HC, *n* = 24), and PT as a control (PD, *n* = 37; HC, *n* = 37). (**C**) Average percentage of cytokine of total response (i.e., IFN-γ/sum of IFN-γ/IL-5/IL-10) between HC and PD across all neuroantigens tested (PINK1, PARKIN, OGDH, GBA, SOD1, and LRRK2). Two-way ANOVA with Dunnett’s test.

PBMCs were processed from whole-blood donations provided by individuals with PD (*n* = 39) and HCs (*n* = 39). Participants of the study were recruited from 3 sites across the US: New York (Columbia University Irving Medical Center [CUMC]), Illinois (Shirley Ryan AbilityLab/Northwestern University), and California (UCSD and La Jolla Institute for Immunology [LJI]) as well as from the Parkinson’s Progression Markers Initiative (PPMI) consortium. Detailed cohort demographics, including the clinical characteristics of the PD cohort, can be found in Supplemental Table 1 (supplemental material available online with this article; https://doi.org/10.1172/JCI180478DS1). We tested 6 PD-related proteins as potential targets of T cell recognition in individuals with PD. These proteins were selected based on their genetic link to PD, their presence in Lewy bodies, and/or their being implicated in preclinical models of PD: PINK1 (4, 22, 23, 28, 29), parkin (5, 22, 23), OGDH (22, 30), GBA (6, 8, 31), SOD1 (25, 32), and LRRK2 (7, 29) (Figure 1A).

To determine whether T cells recognize these PD-related proteins, we assayed pools of 15 amino acid peptides overlapping by 10 residues and spanning the full sequence of each protein: PINK1 (117 peptides), parkin (94 peptides), OGDH (203 peptides), GBA (106 peptides), SOD1 (34 peptides), and peptides predicted to bind HLA class II alleles for LRRK2 (80 peptides). Individual peptide sequences and more detailed pool information can be found in Supplemental Table 2. As a control, we also included a previously described peptide pool directed toward *Bordetella* *pertussis* vaccine antigens (33) (PT, Supplemental Table 2), which individuals are exposed to through Tdap vaccination. We hypothesized that there would be a higher magnitude of T cell–specific responses against these antigens in individuals with PD compared with age-matched HCs. PBMCs from the PD and HC cohorts were stimulated in vitro with the different peptide pools, each composed of peptides derived from a single protein, for 14 days. At the end of the restimulation period, expanded cultures were assayed by tri-color Fluorospot, measuring IFN-γ, IL-5, and IL-10 (Figure 1A), which were selected as representative cytokines associated with Th1, Th2, and Treg responses, respectively.

### Higher PINK1-specific T cell reactivity in PD patients compared with controls.

The T cell reactivity to the neuroantigen pools is shown in Figure 1B. A significant increase in the frequency (the number of samples that responded versus those that did not respond to the peptide pools) of PINK1 reactivity was observed among PD patients compared with HCs, although the magnitude of the PINK1 response was not significantly increased (1.8-fold increase, Fisher’s exact 2-tailed test, *P* = 0.049; 2-tailed Mann-Whitney, *P* = 0.063). There was also a significantly higher number of patients with PD who responded to PT (Fisher’s exact 2-tailed test, *P* = 0.040), when the total response was considered.

The response magnitude of the PD patients for GBA was 2.3-fold higher than the HC cohort, but did not reach statistical significance: there was a 2.3-fold increase (2-tailed Mann-Whitney *P* = 0.054, Fisher’s *P* = 0.073).

The individual cytokine profiles were highly polyfunctional. When the response to all neuroantigens was considered in aggregate, IFN-γ accounted for 33.2% of the total, IL-5 for 44%, and IL-10 for 22.9% in PD patients with a profile similar to HCs (Figure 1C). IL-5 responses were significantly more prevalent than IFN-γ for PD (*P* = 0.0106, 1-way ANOVA with Dunnett’s test). For both cohorts, IFN-γ and IL-5 were significantly higher than IL-10 (*P* = 0.0343 and *P* = 0.0214, respectively, for HC and *P* = 0.0132 and *P* < 0.0001 for PD, 1-way ANOVA with Dunnett’s test). Detailed results for the individual cytokines compared between HC and PD participants are in Supplemental Figure 1, A–C. The difference in PINK1-specific responses between patients with PD and HC was primarily driven by IL-5 production, including both the magnitude of the response (2-tailed Mann-Whitney test, *P* = 0.018) and the number of individuals responding (2-tailed Fisher’s exact test, *P* = 0.035) (Supplemental Figure 1B). The background was subtracted from the protein-specific responses, but to provide insight into background magnitudes, this figure also shows the magnitude of response in DMSO control samples. For IFN-γ and IL-5, the background was around 10 spot-forming cells (SFC) per million PBMCs, for IL-10 it was around 100 SFC. In conclusion, PINK1-specific T cell responses and potentially additional neuroantigen-specific responses, such as GBA, are higher in PD patients than in HCs.

### T cell reactivity in PD is not associated with early time points or other clinical characteristics.

We examined the correlation between neuroantigen-specific T cell reactivity and disease status, including age, time from diagnosis, cognitive function (the Montreal Cognitive Assessment [MoCA]) (34), motor examination (part III from the Unified Parkinson’s Disease Rating Scale [UPDRS]) (35), and medication (levodopa equivalent dose [LED]) (36) scores. We found a positive correlation between age and T cell reactivity to Parkin, a negative correlation between time since diagnosis (years) and T cell reactivity to SOD1, between LED and T cell reactivity to SOD1, and between UPDRS part III and GBA (Supplemental Figure 2). No correlations between these parameters and T cell reactivity to PINK1 were found. We previously found that the α-syn–specific T cell reactivity was higher closer to PD diagnosis and then waned (15), similar to the observations found here for GBA and SOD1. Thus, neuroantigen-specific T cell reactivity is complex, and the responses to the candidate antigens are differentially affected by age and time from diagnosis.

Not all patients with PD have α-syn–specific T cell reactivity (15, 16). To determine whether patients with PINK1-specific T cells also have α-syn–specific T cell reactivity, we correlated the total magnitude of reactivity against PINK1 with the total magnitude against α-syn (*n* = 53 participants that responded to either PINK1 or α-syn, Supplemental Figure 3). There was no correlation between reactivity for the 2 proteins and no significant overlap in response to both antigens (2-tailed Fisher’s exact test, *P* = 0.168).

### Neuroantigen-specific T cell responses as a function of biological sex.

**Figure 2:**
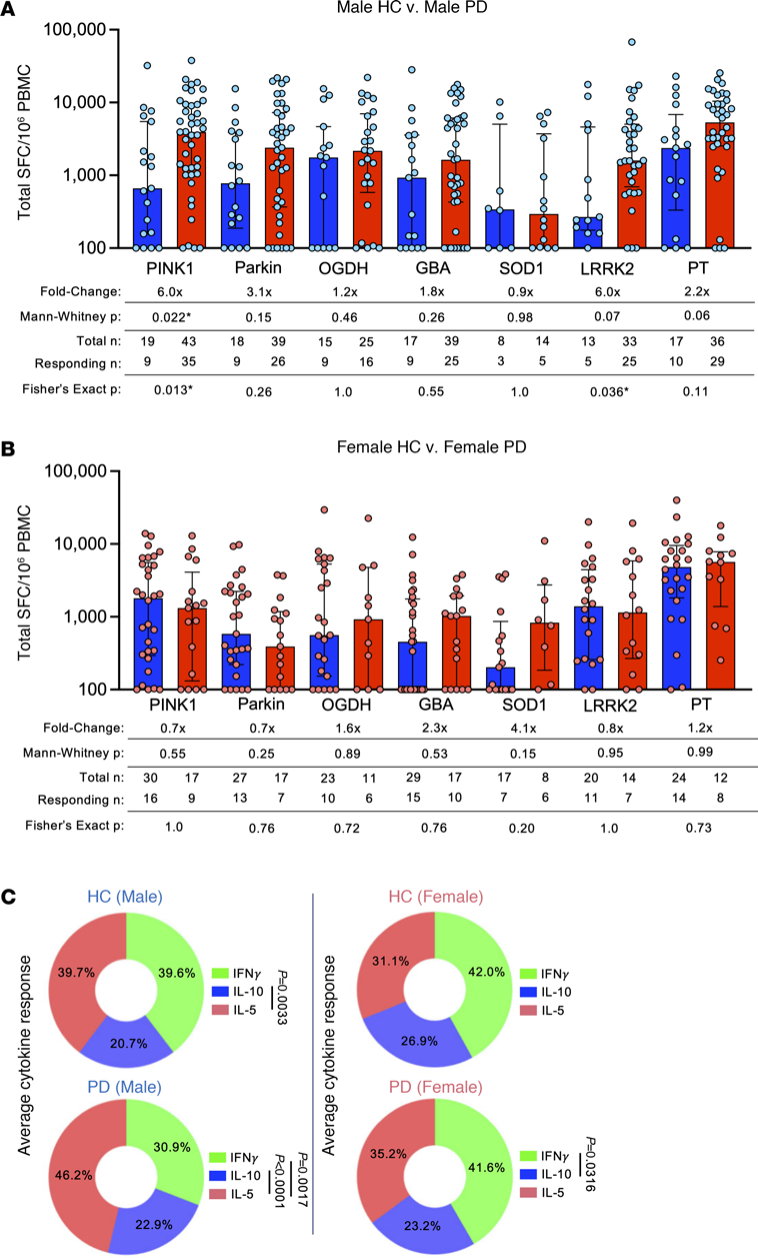
Neuroantigen-specific T cell responses as a function of biological sex. Magnitude of total cytokine response (sum of IFN-γ, IL-5, and IL-10) to neuroantigens and control PT between (**A**) male HCs and PD; (**B**) and female HCs and PD. HCs (blue bars) and PD (red bars); each circle represents an individual participant. Median ± interquartile range displayed. Fold-change is in comparison to HC response. Two-tailed Mann-Whitney *U* tests were performed between HC and PD antigen-cytokine values. Two-tailed Fisher’s exact tests were performed using the geometric mean of the HC group for each individual antigen as a cutoff for the test. (**C**) Average percentage cytokine of total response (i.e., IFN-γ/sum of IFN-γ/IL-5/IL-10) between HCs and PD across all neuroantigens tested (PINK1, PARKIN, OGDH, GBA, SOD1, and LRRK2). Two-way ANOVA with Dunnett’s test.

It is well established that the incidence of PD is higher in males than females (37). We observed that the increased PINK1 response in PD appeared predominantly driven by differences in males (Figure 2, A and B) with a 6.0-fold increase of PD versus HCs in males (2-tailed Mann-Whitney, *P* = 0.022; 2-tailed Fisher’s exact, *P* = 0.013) compared with a 0.7-fold difference in female PD versus HCs (Mann-Whitney, *P* = 0.55; Fisher’s exact, *P* = 1.0). Similarly, LRRK2 responses had more male PD patients than male HCs responding to it, albeit with no significant difference in magnitude of response (6.0-fold, Mann-Whitney *U* test, *P* = 0.07; Fisher’s exact test, *P* = 0.036) versus females (0.8-fold difference in PD versus HCs; Mann-Whitney *U* test, *P* = 0.95; Fisher’s exact test, *P* = 1.0).

When the total responses of male versus female participants were broken down into their individual cytokine constituents, we observed comparable responses as reported above for the entire cohort (Figure 1C). IL-5 was the most prominent cytokine produced against all antigens in males (39.7% male HCs, 46.2% male PD) and was not significantly different from females (31.1% female HCs, 35.2% in female PD; Figure 2C). IFN-γ accounted for 39.6% and 30.9% of the cytokine response in male HCs and PD, and in female HCs and PD, accounted for 42.0% and 41.6%, respectively. The IL-10 response in male HCs and PD response was 20.7% and 22.9%, respectively, compared with female HCs and PD (26.9% and 23.2%, Figure 2C). Intriguingly, it appears that there was an IFN-γ bias toward neuroantigens in both female HCs and PD compared with males. As in female PD, but not male, IFN-γ response was significantly higher than IL-10 (*P* = 0.0316, 1-way ANOVA with Dunnett’s test). In males, IL-5 was significantly higher than IL-10 for both HCs and PD (*P* = 0.0033 and *P* < 0.0001, respectively) and was significantly higher than IFN-γ in males with PD (*P* = 0.0017; 1-way ANOVA with Dunnett’s test). Additional detailed individual antigen/cytokine differences between male and female HCs and PD can be found in Supplemental Figure 4. Male patients with PD had significantly higher magnitude and frequency of PINK1-specific T cell responses for both IFN-γ and IL-5 (IFN-γ; fold change 3.3×, 2-tailed Mann-Whitney *U* test, *P* = 0.009; 2-tailed Fisher’s exact test, 0.018, IL-5; fold change 8.4, 2-tailed Mann-Whitney *U* test, *P* = 0.020; Fisher’s exact test, 0.03; Supplemental Figure 4, A and C). In contrast, responses comparing female participants were remarkably similar. Taken together, these results suggest that reactivity to different PD autoantigens exhibits a sex bias in terms of specific antigen reactivity and the types of cytokines produced in response.

### Phenotypic characterization of PINK1-responsive T cells.

**Figure 3:**
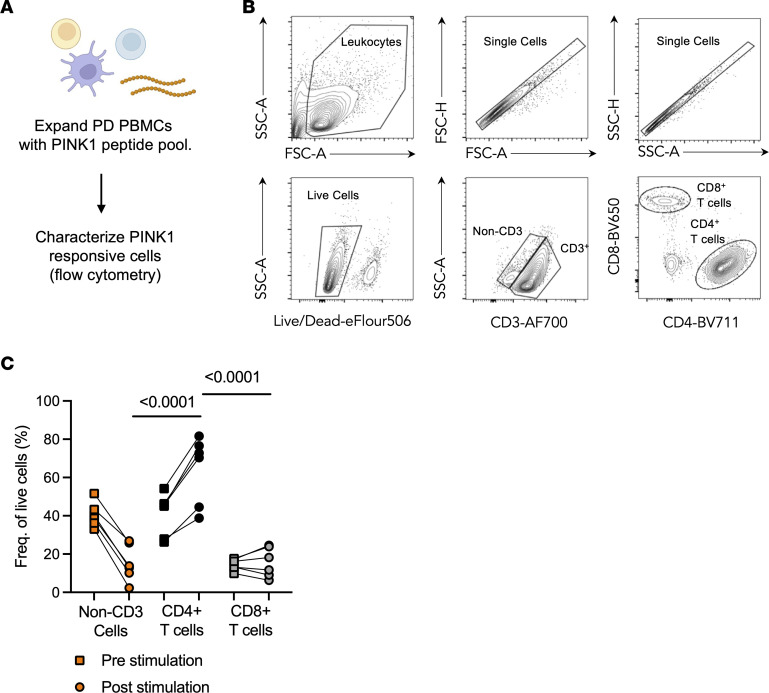
Phenotypic characterization of PINK1-responsive T cells. (**A**) Diagram describing experimental design to characterize PINK1-expanded PBMC cultures from individuals with PD. (**B**) Representative gating strategy depicting the identification and quantification of live, singlet, non-CD3^+^ or CD3^+^, CD4^+^/CD8^+^ T cells. (**C**) Frequency of live cells of CD4 (black), CD8 (gray), and non-CD3 (orange) from before stimulation (squares) and after PINK1-stimulated PD PBMCs (circles). Two-way ANOVA with Tukey’s multiple comparisons for post-stimulation samples.

We then characterized in more detail the phenotype of the expanding/cytokine-producing cells in cultures stimulated with the PINK1 peptide pool and compared them to the nonexpanded cell subset frequencies. We did this for a subset of individuals for which we had enough PBMCs available, as PBMCs were prioritized for the identification of individual PINK1 epitopes (below). To do so, before and after in vitro expansion, we analyzed PBMC cultures from a subset of PD participants (*n* = 6, 5 males, 1 female) either unstimulated (nonexpanded) or stimulated with the PINK1 peptide pool by flow cytometry (Figure 3A). Gating on live, single, CD3^+^ cells (Figure 3B), the predominant cell type in the PINK1 expanded cultures were CD4^+^ T cells (64% ± 18% of live cells, Figure 3C), which all increased following expansion and were present in significantly higher frequencies than CD8^+^ T cells (16% ± 8%, *P* < 0.0001, no change after expansion) and non-CD3 cells (15% ± 9%, *P* < 0.0001, all of which decreased after expansion). The nonexpanded frequency of these cell populations matched what has been previously described for PBMC samples (38). These data demonstrate that the predominant cell type recognizing the PINK1 epitopes is CD4^+^ T cells, consistent with reports for other PD neuroantigens (15, 16, 39).

### Identification of individual PINK1 epitopes eliciting T cell responses in PD.

**Figure 4:**
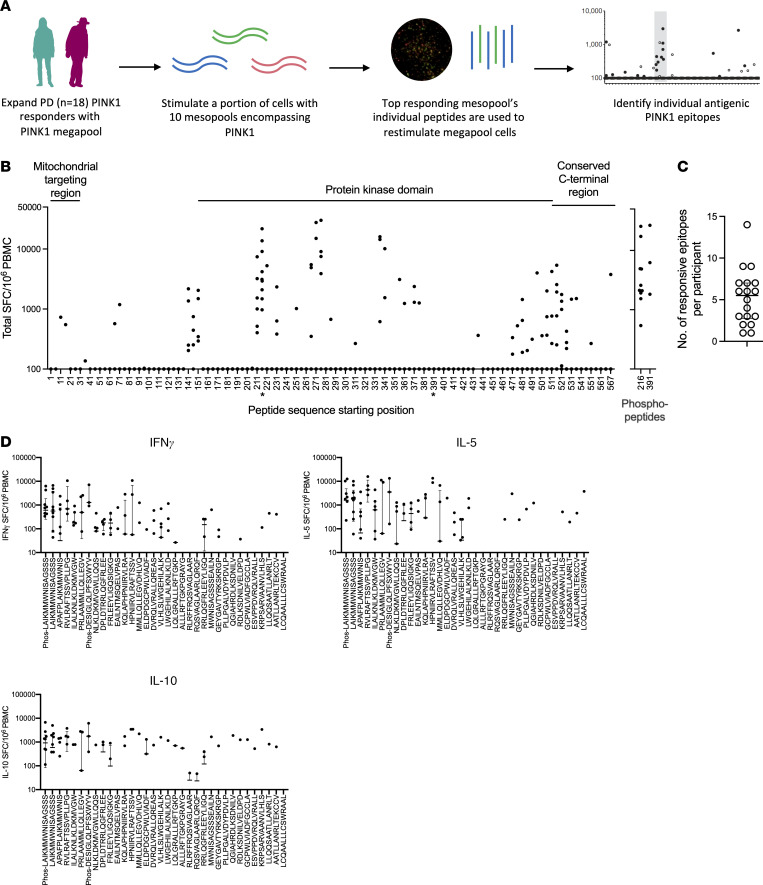
Identification of PINK1 epitopes eliciting T cell responses in PD. (**A**) Experimental design utilized to identify PINK1 epitopes. PINK1 megapool was used to expand previously identified PD PINK1 responders. A portion of megapool expanded cells for each participant were then restimulated with 10 PINK1 mesopools (smaller pools containing, on average, 12 individual PINK1 epitopes). The individual epitopes from the top 3 responding mesopools for each participant were then used to restimulate the remaining megapool expanded cells, allowing for the identification of individual antigenic PINK1 epitopes. (**B**) Individual PINK1 epitope responses (total cytokine, sum of IFN-γ, IL-5, IL-10) displayed in relation to the major regions of the PINK1 protein (left to right across amino acid 1–581; * at the x-axis for the peptide sequence indicates peptides that were also included as phosphorylated versions). The right graph displays phosphorylated peptides. Each dot is a participant/peptide combination. The response was considered positive when 3 criteria were met: (a) background-subtracted SFC/million cells above or equal to 100 SFC, (b) a fold-change of 2 or more compared with the negative control, and (c) a significant *P* value comparing triplicates for the negative control to the test triplicate. (**C**) Number of individual epitopes recognized by each of the 18 individual PD participants tested. (**D**) Individual cytokine responses (IFN-γ, IL-5, and IL-10) toward the 34 PINK1 epitopes displayed in order of frequency of recognition. Median ± interquartile range is shown.

To identify individual PINK1 epitopes, we stimulated a subset (*n* = 18; 15 male and 3 female) of previously identified PD PINK1 responders with the pool of PINK1 overlapping peptides. The resulting cultures were restimulated with 10 separate PINK1 “mesopools” (smaller pools of ~12 individual peptides) that spanned the PINK1 protein. The top 3 highest mesopool responses for each participant were then deconvoluted to identify individual PINK1 epitopes (Figure 4A). All peptides were tested individually in the deconvolution experiments, which identified distinct epitopes, and for all measures shown, data points for each individual participant are included. We identified 34 individual peptides that elicit T cell responses in PD patients (Figure 4B and Supplemental Table 2). The average number of PINK1 epitopes recognized by each PD patient was 5.2 (median of 5.5, range 1-14, Figure 4C), thereby addressing whether the same participant responds to several epitopes or if the response is heterogeneous in the participants. The dominant epitope, aa216 LAIKMMWNISAGSSS, was recognized by 55.5% of the PD patients (Table 1). The next most recognized epitope, aa220 APAFPLAIKMMWNIS, was recognized in 27.7% of PD patients. A total of 7 epitopes were recognized in 3 or more participants. Of the 3 female PD patients included in these experiments, 2/3 had T cell responses against the dominant aa216 LAIKMMWNISAGSSS epitope, while the remaining participant’s dominant epitope was aa511 LWGEHILALKNLKLD (also observed in a male participant).

Individual IFN-γ, IL-5, and IL-10 responses toward the 34 identified PINK1 epitopes showed a similar pattern of responses as the original PINK1 megapool, with all 3 cytokines represented (Figure 4D). Of note, the most commonly recognized epitope (a.a.216LAIKMMWNISAGSSS) typically elicited responses with all 3 cytokines, with some participants producing all 3 cytokines against the epitope, while others produced 1 or 2. As expected, some more unique, single-participant responsive epitopes only resulted in 1 specific cytokine (IL-5 in the case of aa567 LCQAALLLCSWRAAL).

### Determination of potential HLA restriction of PINK1 epitopes.

The results above indicate that CD4^+^ T cells are the predominant T cell subset expanded following PINK1 peptide pool stimulation. To infer potential HLA restrictions, we examined each epitope recognized in 2 or more participants, following an approach outlined previously (40), and the NetMHCIIpan EL 4.1 tool hosted in the Immune Epitope Database (IEDB) analysis resource. Inferred restrictions indicated by the corresponding β chain are summarized in Table 2. For each restriction, the number of participants responding to the epitope that expresses the β chain allele is indicated in parentheses; alleles present in 2 or more participants who responded to the epitope are highlighted in bold.

At least 1 restriction element was predicted for 14 of the 18 epitopes recognized in multiple participants. The epitopes spanning residues 216–230, in their WT and phosphorylated forms, were recognized in 9 and 10 participants, respectively, and were associated with 13 different HLA class II alleles. The most restrictions were associated with DRB1*15:01 and DRB4*01:01, which are predicted to restrict 7 and 6 of the epitopes, respectively. For the remaining 4 epitopes, no HLA class II restrictions were inferred. Still, in those cases, the 15-mers were found to contain 9-mer/10-mer peptides predicted to bind to HLA class I molecules expressed in the responding participant (italicized in Table 2), consistent with CD8^+^ T cells corresponding to a minor fraction of the T cell expanded by the in vitro culture. In conclusion, multiple epitopes show promiscuous HLA binding and have several possible HLA restrictions.

## Discussion

Identifying the specific antigenic targets recognized by T cells is essential for understanding the pathogenesis of infectious disease (41, 42) and autoimmunity (43, 44). In disorders with autoimmune features, antigen identification is important for determining the basis of vulnerable cell populations, sources of antigenic substrates, and potential immune biomarkers related to the disease. In PD, a key topic of interest has been the identification of the roles that T cells play in the development and/or progression of PD neurodegeneration. Our previous work (15, 16) and the work of others (17, 45) have shown that α-syn is a target of peripheral T cell responses in some PD patients. However, not all PD patients possess these autoinflammatory T cells, and for those who do, their frequency wanes over the course of the disease (15). We have also previously shown that τ is recognized by T cells broadly in the population irrespective of age and disease status (39).

Proteins related to neurodegenerative diseases have long been examined for their possible roles in pathogenesis, and particularly recently, their roles have been expanded into nonneuronal populations such as glia, microglia (21, 46, 47), and peripheral immune cells (14, 21, 48, 49). Studies in mouse models of PD have implicated mitochondrial proteins as potential antigens, particularly the mitochondrial matrix protein, OGDH, which is implicated in the autoimmunity-linked disorder primary biliary cholangitis (22, 50). This, in turn, could be linked to the PINK1-parkin interactions that have been considered to control the turnover of damaged mitochondria in macroautophagy (51, 52) or via the formation of mitochondria-derived vesicles that can elicit a process that has been called “mitochondrial antigen presentation” (53).

Here, we tested whether OGDH and other proteins involved in the PD disease process (i.e., PINK1, PARKIN, GBA, SOD1, LRRK2) may elicit T cell responses from individuals with PD and identified the mitochondria-associated protein PINK1 as an autoantigen recognized by T cells from PD patients. The PINK1-specific T cell responses were predominantly detected in male PD patients compared with female patients. The increased incidence of PD among males is well established (54), while more recently, distinct differences in the clinical phenotype, progression, and therapeutic treatment between biological sexes have been appreciated (55). Such sex-based distinctions appear to extend to the immune system of PD, with the monocyte profile in females with PD being more inflammatory than males with PD (56). Additionally, levels of plasma cytokines have been found to differ between males and females, with substantially increased IL-4 and IL-10 levels in males with PD (57). In this study, we expand on this sex-specific immune profile with our observation of male-driven PINK1 T cell responses, as well as an IFN-γ bias among females in T cell reactivity toward the tested neuroantigens. These differences may be driven by the known hormonal, genetic, and environmental factors previously shown to be influenced by biological sex in the pathobiology of PD. Future studies should analyze the sex bias regarding T cell reactivity to some of these antigens beyond the current correlative results. It will be, for example, informative to analyze the dynamics of cell reactivity longitudinally to determine the dynamics of T cell reactivity in the different sexes as a function of disease progression and in the prodromal stages, preceding diagnosis and symptom onset. In particular, we are interested in determining, in more detail, the transcriptomic and epigenetic profiles associated with the responses as a function of sex to examine the hypotheses that responses to different antigens might have different effects (either proinflammatory or regulatory). Other hypotheses that could be investigated are related to sex differences in antigen expression or the development of antigen-specific tolerance.

The participants varied in terms of time after diagnosis, with an average of 6 years, representing a relatively short time frame for PD. Within this short time frame, PINK1-specific T cell reactivity did not correlate with the time since diagnosis. We have previously found a correlation between α-syn–specific T cell reactivity and the time after diagnosis, with higher reactivity detected closer to the time of diagnosis (15). However, the correlation with time since diagnosis should be considered cautiously since a large diversity of evolution may occur depending on whether the diagnosis was made early or late following symptom onset.

We identified 34 individual PINK1 epitopes responsible for mediating most of the PINK1-specific T cell response. PINK1 contains 4 major domains: the mitochondrial targeting region (aa 1–76), a transmembrane segment (aa 95–111), a protein kinase domain (aa 156–511), and a conserved C-terminal region (aa 517–581). Interestingly, we observed “regions” of reactivity in PINK1 (similar to what can be observed in pathogen (33, 42) and autoimmune (58, 59) antigens, including α-syn (16)), with distinct clusters of antigenicity in both the protein kinase domain and the conserved C-terminal region. There was no evidence for mimicry based on the lack of overlap of epitope sequences in the IEDB. The protein kinase domain contained the most commonly reactive PINK1 epitope aa216 LAIKMMWNISAGSSS and its phosphorylated version (phosphorylated serine at aa228), which were generally both recognized by T cells from the same participant. Ser-228 is a key regulatory phosphorylation site in the kinase catalytic activity of PINK1 (60). Interestingly, the other phospho-antigen we tested, aa391 DESIGLQLPFSXWYV (Ser-402) located in the C-terminal domain, was also reactive but not its unphosphorylated counterpart. Ser-402, like Ser-228, is a key regulatory site for the kinase activity of PINK1 (60). A limitation of our study is that truncated or altered peptides can act as TCR agonists or antagonists, and using a large number of peptides can result in competition for HLA binding. For this reason, future studies can determine the optimal size and frame of the identified epitopes in more detail, thus addressing the agonist concern. The possibility that altered peptides might lead to false negative results must be considered and cannot be excluded. We hypothesize that PINK1 may be recognized as an autoantigen in PD because, similar to α-syn, it can be found within Lewy bodies (28, 29) and thus is potentially phagocytosed and presented by either microglia or other CNS antigen-presenting cells to T cells (61).

It would be of interest to obtain information regarding dose response and affinity of responding epitope-specific T cells, but this is not straightforward, as a dose-response experiment would need to be performed for the 14-day stimulation, requiring cell numbers beyond what is available. Future studies could derive short-term T cell lines and/or clonal populations. This strategy would also allow determination of the TCR repertoire of the antigen-specific T cells and the ability to track these cells in single-cell datasets and determine their phenotype in more depth. This would also allow us to study both CD4^+^ and CD8^+^ T cells in more detail, since CD8^+^ T cells may also play a role in disease progression.

As deficiencies in PINK1 and parkin activity lead to mitochondrial antigen presentation in mouse models (22), it is interesting to speculate on how this could occur in PD. PINK1 is thought to be constantly produced by local translation in axonal mitochondria and be tethered to mitochondria by the proteins synaptojanin 2 and synaptojanin 2 binding protein (52). If PINK1 is not continuously degraded, it may overstabilize parkin and block the normal mitochondrial turnover (62). It may be that PINK1, which is not normally turned over by the proteasome, macroautophagy, or chaperone-mediated autophagy, produces antigenic epitopes, including peptides with posttranslational modifications such as phosphorylated residues. If the resulting PINK1-derived peptides can act as “neoantigens” that are not recognized as “self” they may activate T cell responses, a feature that occurs with immune responses to synucleins (61). Broadly, a role for altered protein degradation for PINK1 would be analogous to the blockade of protein degradation for other PD-related proteins, including α-syn (63) and modified α-syn (64), LRRK2 (65), and GBA (66).

We identified predicted HLA restrictions for the most recognized PINK1 epitopes by PD patients. Interestingly, most restrictions were associated with the DRB1*15:01 and DRB4*01:01 alleles. DRB1*15:01 was previously found to be more common in the PD population by our group and capable of presenting certain α-syn T cell epitopes (16), was found to be associated with PD in a GWAS study (67), and is also associated with increased Alzheimer’s disease risk (68). DRB4*01:01, to our knowledge, has not been linked to PD before, but is present in a high-risk haplotype associated with type 1 diabetes (69). While these are 2 of the most common HLA class II specificities in the general worldwide population, it is notable that they are not linked, with DRB1*15 alleles almost invariably associated with DRB5 alleles, and DRB4 generally associated with DRB1*04, 07, and 09. DRB1*01:02, DRB1*04:04, and DRB3*02:02 were predicted to be the next most frequently utilized alleles, associated with 4 epitopes each.

Moreover, the cytokines we observed responding to the neuroantigens tested (including PINK1) represent a potential multifaceted immunological phenotype with both pro- and antiinflammatory cell types at play. PD is a heterogeneous disease (70), and it is possible that the specific antigens recognized and cytokines produced are due to this underlying heterogeneity in the patient population. Furthermore, the T cell reactivity against PINK1 does not correlate in terms of magnitude or frequency of responses with α-syn–specific responses, further providing evidence for heterogeneity of T cell responses in PD.

In conclusion, our study has identified PINK1 as a common autoantigenic target of T cells in PD. These responses are predominantly associated with male PD individuals, multiple secreted cytokines toward PINK1 were observed, and specific epitopes and corresponding restricting HLA alleles are reported. These results reinforce the need for studying PD in the context of the immune system, with the goal of developing personalized immune-based therapies.

## Methods

### Sex as a biological variable.

Our study included both male and female participants (Supplemental Table 1). The results have been reported as an aggregate for the entire cohort, and additionally, with female and male participants analyzed separately.

### Study participants.

Subjects with idiopathic PD (*n* = 39) and HCs (*n* = 39) were recruited by the Movement Disorders Clinic at the Department of Neurology at CUMC, by the clinical core at LJI, by the Parkinson and Other Movement Disorder Center at UCSD, and by the movement disorder specialists at the Parkinson’s disease and Movement Disorders program at Shirley Ryan Ability Lab. For these participants, no information regarding whether they harbor any known variants/mutations is available. The PPMI (https://dx.doi.org/10.17504/protocols.io.n92ldmw6ol5b/v2) also recruited subjects with PD (*n* = 21) and HCs (*n* = 10). Among these 21 participants with PD, 13 had sporadic PD (62%), 5 carry variants of LRRK2, 2 carry variants of GBA, and 1 participant carries a Parkin variant. Inclusion criteria for PD patients consisted of (a) clinically diagnosed PD with the presence of bradykinesia and either resting tremor or rigidity, (b) PD diagnosis between ages 35–80, (c) history establishing dopaminergic medication benefit, and (d) ability to provide informed consent. Exclusion criteria for PD were atypical parkinsonism or other neurological disorders, history of cancer within past 3 years, autoimmune disease, and chronic immune modulatory therapy. Age-matched HCs were selected on the basis of (a) age 45–85 and (b) ability to provide informed consent. Exclusion criteria for HCs were the same as PD except for the addition of self-reported PD genetic risk factors (i.e., PD in first-degree blood relative). For the LJI cohort, PD was self-reported. The recruited individuals with PD all met the UK Parkinson’s Disease Society Brain Bank criteria for PD. Cohort characteristics are shown in Supplemental Table 1.

For a subset of participants, specific clinical information was collected. The time since PD diagnosis is the time of donation following the initial diagnosis of PD in years to understand the duration of the disease. The Movement Disorder Society–UPDRS (MDS-UPDRS) is a standard scale for PD symptoms, with higher numbers reflecting more debilitating symptoms. Part III reflects the motor symptoms. The MoCA score is a standard test for cognitive impairment, with lower scores representing greater impairment and normal cognition at 26–20 points. The LED is a calculation that estimates the effective amount of L-DOPA available to the patient and is intended to provide a comparison between different formulations (regimen, dosage, and timing), with higher numbers indicating higher amounts administered, and is in units of milligrams.

### PBMC isolation.

Venous blood was collected from each participant in either heparin or EDTA containing blood bags or tubes. PBMCs were isolated from whole blood by density gradient centrifugation using Ficoll-Paque Plus (GE 17144003). In brief, blood was first spun at 803*g* for 15 minutes with brakes off to remove plasma. Plasma-depleted blood was then diluted with RPMI, and 35 mL of blood was carefully layered on tubes containing 15 mL Ficoll-Paque Plus. These tubes were then centrifuged at 803*g* for 25 minutes with the brakes off. The interphase cell layer resulting from this spin were collected, washed with RPMI, counted, and cryopreserved in 90% v/v FBS and 10% v/v dimethyl sulfoxide (DMSO) and stored in liquid nitrogen until tested. The detailed protocol for PBMC isolation can be found at protocols.io (https://dx.doi.org/10.17504/protocols.io.bw2ipgce).

### Antigen pools.

For antigen candidates smaller than 1,100 amino acids, 15-mer peptides overlapping by 10 amino acids spanning the entire protein were used: PINK1 (115 peptides; UniProt ID Q9BXM7), parkin (91 peptides; O60260), OGDH (203 peptides; Q02218), GBA (106 peptides; P04062), and SOD1 (29 peptides; P00441). This approach was used as it is common practice in epitope discovery efforts, which allows us to stimulate cell cultures with equimolar amounts of peptides reproducibly (71). For LRRK2 (2527 aa in length; Q5S007), we predicted binding to HLA class II alleles using the 7-allele method (72) and selected the top 80 peptides with a median percentile score below 20. We also included peptides with a phosphorylated serine for PINK1 (aa228 and aa402), PARKIN (aa65), and SOD1 (aa99, 103, 106, and 108). For the pertussis peptide pool, we used a previously defined and characterized pool of 132 peptides (73). Peptides were synthesized commercially as crude material on a 1 mg scale by TC Peptide Lab. Lyophilized peptide products were dissolved in 100% (DMSO) at a concentration of 20 mg/mL, and their quality was spot checked by mass spectrometry. The purity is greater than 85% for more than 85% of the peptides. Overlapping and predicted class II peptides were combined to form antigen pools for all the antigens tested, always keeping individual antigens in separate pools. Our lab routinely identifies CD4^+^ and CD8^+^ T cell epitopes, as well as uses existing data in the Immune Epitope Database and Analysis Resource (IEDB; ref. 74) to develop peptide “megapools” (71). A detailed protocol for making megapools is found in the open-access publication by da Silva Antunes, et al. (71) The utilization of these megapools allows the ability to test a large number of epitopes spanning multiple HLA types. Specific sequence identities and reference numbers making up the various antigen pools used in this study can be found in Supplemental Table 2.

### In vitro expansion of antigen-specific cells and Fluorospot Assay.

In vitro expansion and subsequent FluoroSpot assay were performed as previously described (14, 15). Briefly, PBMCs were thawed and then stimulated with neuroantigen or PT peptide pools (5 μg/mL) for 4 days. After 4 days, cells were supplemented with fresh RPMI and IL-2 (10 U/mL, ProSpec Bio) and fed again every 3 days, as described in detail at https://www.protocols.io/view/pbmc-stimulation-with-peptide-pools-and-fluorospot-bphjmj4n After 2 weeks of culture, T cell responses to neuroantigen pools were measured by IFN-γ, IL-5, and IL-10 FluoroSpot assay (Mabtech FSP-010708). Plates (Mabtech) were coated overnight at 4°C with an antibody mixture of mouse anti-human IFN-γ (clone 1-D1K), mouse anti-human IL-5 (clone TRFK5), and mouse anti-human IL-10 (clone 9D7), all from Mabtech. 1 × 10^5^ harvested cells were plated in each well of the coated Fluorospot plots along with each respective antigen (5 μg/mL) and incubated at 37°C in 5% CO_2_ for 22 hours. Cells were also stimulated with 10 μg/mL phytohemagglutinin (PHA) (positive control) as well as DMSO (negative control) to assess nonspecific cytokine production. All conditions were tested in triplicate. After incubation, cells were removed and membranes were washed. An antibody cocktail containing IFN-γ (7-B6-1-FS-BAM), IL-5 (5A10-WASP), and IL-10 (12G8-biotin), all from Mabtech, prepared in PBS with 0.1% BSA was added and incubated for 2 hours at room temperature. Membranes were then washed again, and secondary antibodies (anti-BAM-490, anti-WASP-640, and SA-550, all from Mabtech) were then incubated for 1 hour at room temperature. Lastly, membranes were washed, incubated with fluorescence enhancer (Mabtech), and air-dried for reading. Spots were read and counted using the Mabtech IRIS system. Responses were considered positive if they met all 3 criteria: (a) DMSO background subtracted spot forming cells per 10^6^ were 100 or greater, (b) stimulation index 2 or more compared with DMSO controls, (c) *P* ≤ 0.05 by Student’s t test or Poisson distribution test. The detailed protocol for the Fluorospot assay can be found at https://www.protocols.io/view/fluorospot-assay-bpspmndn

### Flow cytometry.

In vitro expanded cells were washed, counted, and plated in a 96-well plate at a density of 1 × 10^6^ cells/well. Cells were then stained with a mixture of the following antibodies: Fixable Viability Dye eFluor 506 (Thermo Fisher), CD3-AF700 (BD, RRID:AB_10597906), CD4-BV711 (BD, RRID:AB_2740432), and CD8-BV650 (Biolegend, RRID:AB_11125174) for 30 minutes at 4°C in the dark. Stained cells were then washed twice and resuspended in 100 μL PBS to be run on an LSR II flow cytometer (BD; a detailed protocol can be found at https://doi.org/10.17504/protocols.io.bwu9pez6). FCS files produced from the LSR-II were then analyzed using FlowJo, version 10.8.2, software (Tree Star; RRID:SCR_008520; https://www.flowjo.com/solutions/flowjo).

### HLA typing.

Participants were HLA typed at the American Society for Histocompatibility and Immunogenetics–accredited (ASHI-accredited) laboratory at Murdoch University (Western Australia). Typing for class I (HLA A, B, and C) and class II (DQA1, DQB1, DRB1, DRB3, DRB4, DRB5, and DPB1) was performed using locus-specific PCR amplification of genomic DNA. Specific HLA loci were PCR amplified using sample specific multiplex identifier–tagged (MID-tagged) primers that amplify polymorphic exons from class I (A, B, C, exons 2 and 3) and class II (DQA1 and DQB1; exons 2 and 3, DRB and DPB1; exon 2) major histocompatibility complex (MHC) genes relevant to epitope binding and presentation. Therefore, rare alleles that differ by a single nucleotide in exon 1 cannot be excluded due to missing coverage of exon 1. MID-tagged primers were optimized to minimize allele dropouts and primer bias. Amplified DNA products from unique MID-tagged products (up to 96 MIDs) were quantitated, pooled in equimolar ratios, and subjected to library preparation using NEBNext Ultra II Library Prep Kits (New England Biolabs). Libraries were quantified using the Jetseq Library Quantitation Kit (Meridian Bioscience) and High Sensitivity D1000 Screen Tape on an Agilent 2200 Tapestation (Agililent) for concentration and size distribution. Normalized libraries were sequenced on the Illumina MiSeq platform using the MiSeq V3 600-cycle kit (2 × 300 bp reads). Sequences were separated by MID tags, reads were quality-filtered, and alleles were called using an in-house accredited HLA caller software pipeline, minimizing the influence of sequencing errors. Alleles were called using the latest IMGT HLA allele database as the allele reference library. The algorithm was developed in house and relies on periodically updated versions of the freely available international immunogenetics information system (RRID:SCR_012780; imgt.org) and an ASHI-accredited HLA allele caller software pipeline, IIID HLA analysis suite (http://www.iiid.com.au/laboratory-testing/). Sample report integrity was tracked and checked using proprietary and accredited Laboratory Information and Management System (LIMS) and HLA analysis reporting software that performs comprehensive allele balance and contamination checks on the final dataset.

### Prediction of HLA restriction.

Potential HLA class II restrictions were determined on the basis of MHC binding predictions. Predictions were performed using the IEDB’s analysis tools suite (IEDB: RRID:SCR_006604; http://tools.iedb.org/main/tcell/), and the recommended (as of August 2023) NetMHCIIpan algorithm, version 2023.05 EL 4.1 (https://services.healthtech.dtu.dk/services/NetMHCIIpan-4.1). Restrictions were assigned using a predicted binding percentile score threshold of 20% or less. Because HLA typing was unavailable for the DPA (the α locus of the HLA-DP) locus, predicted DPA/B dimer binding was based on known (typically strong) A/B haplotype linkages. Accordingly, DPA1*02:01 was assigned to DPB1*01:01, and DPA1*01:03 to all other DPB1. While both DQA and DQB loci are polymorphic, only haplotype linked (cis) A and B loci are believed to form stable dimers (75, 76). Thus, for DQB1*05 and 06 alleles, only dimers with DQA1*01 were considered when performing prediction analyses, and for DQB1*02, 03, and 04, only dimers with DQA1*02, 03, 04, 05, and 06 were considered. The HLA DRA1 locus is largely monomorphic, with the rare variants mutated outside of the surface exposed domains, and is thus not considered for binding predictions.

### Statistics.

Statistical analyses were performed, and graphs were created using GraphPad Prism’s descriptive statistics, 2-tailed Mann-Whitney tests, 1-way ANOVA with Dunnett’s multiple-comparisons test, 2-tailed Fisher’s exact tests, and Spearman’s r tests as applicable (GraphPad Prism, RRID:SCR_002798, version 9).

### Study approval.

All participants provided written informed consent for participation in the study. Ethical approval was obtained from the Institutional Review Boards at LJI (protocol nos: VD-124 and VD-118), CUMC (protocol number IRB-AAAQ9714 and AAAS1669), UCSD (protocol number 161224), Shirley Ryan Ability Lab/Northwestern University (protocol number STU00209668-MOD0005), and PPMI (protocol number 20216216 and 20200597).

### Data availability.

The data reported within this manuscript are available in the Supporting Data Values file and at Zenodo (https://zenodo.org/records/14227560?preview=1&token=eyJhbGciOiJIUzUxMiJ9.eyJpZCI6IjA3OTNiMWFiLWQ4YWItNDM5Yi05OGM0LWRjYWMxMmNmMmNiNSIsImRhdGEiOnt9LCJyYW5kb20iOiJkNGUzNWNkZjdmZWJjYzQ5NjJhMGY2YzAyN2JmZDJhMSJ9.rBStrqTqv2UwSJLBtQl1bj23cyal8Bdb3GrkYCbRuv_pkcJ8iIsGvYfx2LXkbGp9P5Y5ufHtM-xpvLuJiGEfgQ). Flow cytometry files can be found at https://flowrepository.org/id/FR-FCM-Z7TB with the participant key at Zenodo (https://doi.org/10.5281/zenodo.12701538). PPMI data used in preparation of this article were obtained in January 2024 from the PPMI database (https://www.ppmi-info.org/access-data-specimens/download-data; RRID:SCR_006431). For up-to-date information on the study, visit http://www.ppmi-info.org

## Author contributions

CSLA, AS, and DS participated in the design and direction of the study. GPW, TM, A Freuchet, JS, JRLJ, and CSLA performed and analyzed the experiments. A Frazier, NKT, IL, JGG, and RNA recruited participants and performed clinical evaluations. SAM and EJP performed HLA typing. GPW, A Freuchet, DS, AS, and CSLA wrote the manuscript. All authors read, edited, and approved the manuscript before submission.

## Supplementary Material

Supplemental data

Supporting data values

## Figures and Tables

**Table 1:**
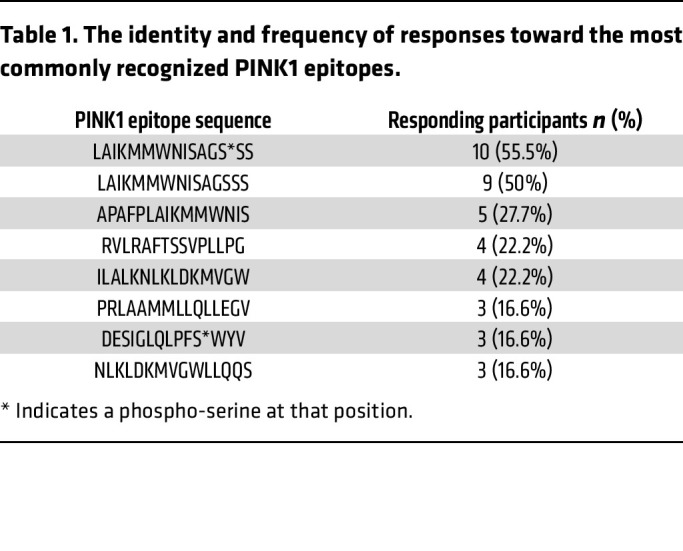
The identity and frequency of responses toward the most commonly recognized PINK1 epitopes.

**Table 2:**
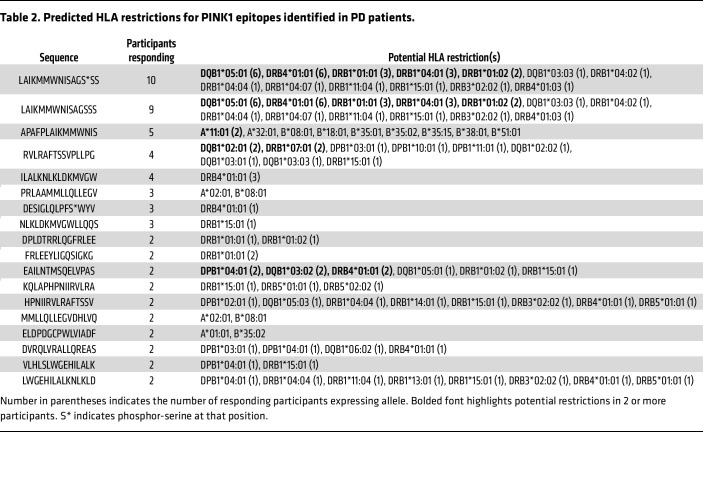
Predicted HLA restrictions for PINK1 epitopes identified in PD patients.

## Version 1 12/17/2024

In-Press Preview

## Version 2 02/17/2025

Electronic publication
