# Supplementary material for: PINK1 is a target of T cell responses in Parkinson’s disease
Source: J Clin Invest. 2024 Dec 17;135(4):e180478. doi: 10.1172/JCI180478 (PMC11827839; doi:10.1172/JCI180478)
Supplement: Supplemental data [file jci-135-180478-s018.pdf]

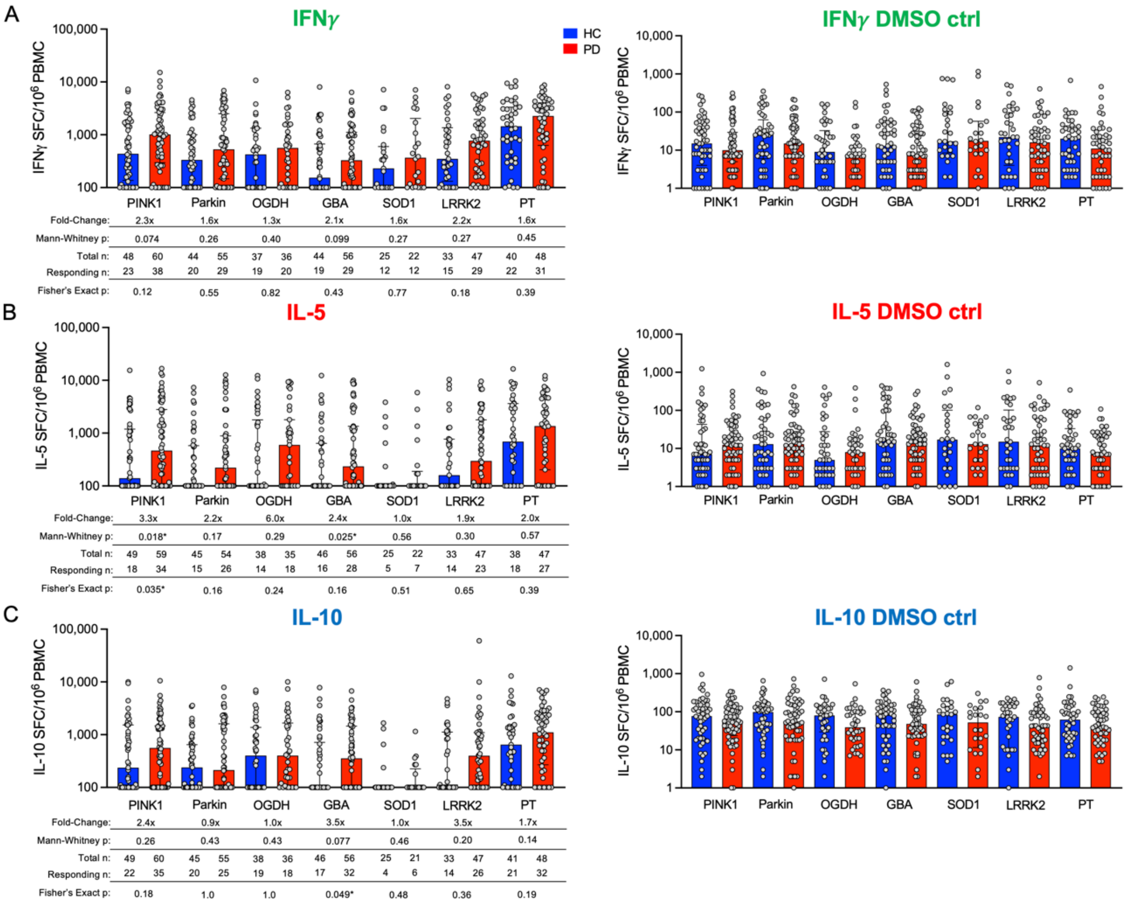

Supplemental Figure 1: Individual cytokine responses towards neuroantigens among PD patients. The magnitude of the individual cytokine response for a) IFNγ, b) IL-5, c) IL-10 by PBMCs from PD and age-matched HC in response to peptide pools (left) and the DMSO control (right). HC (blue bars) and PD (red bars), each circle representing an individual participant. Please note that the y-axis scale is different between left and right. Median ± interquartile range displayed. Fold-change is in comparison to HC response. Two-tailed Mann-Whitney tests were performed between HC and PD antigen-cytokine values. Two-tailed Fisher tests were performed using the geometric mean of the HC group for each individual antigen as a cutoff for the test.

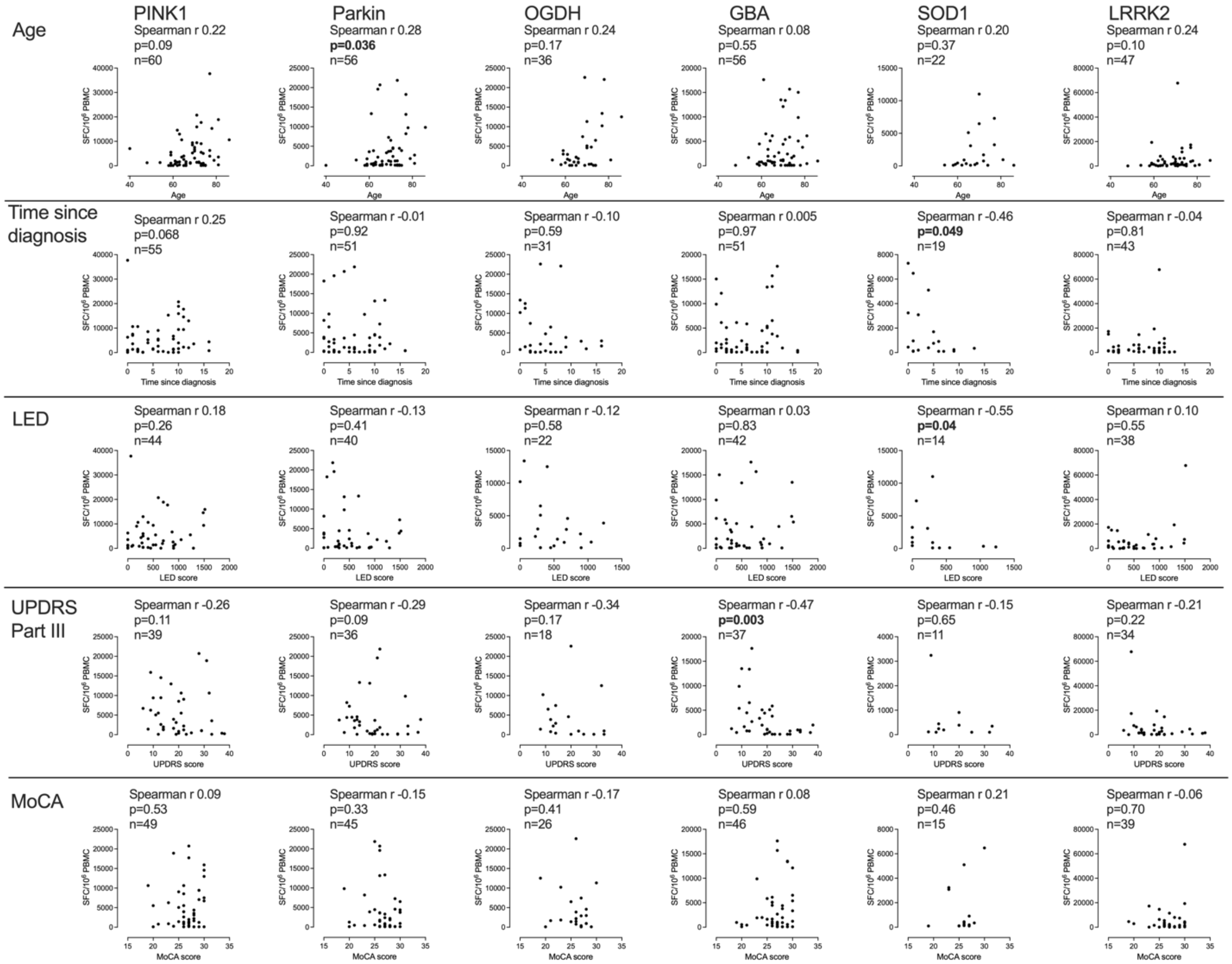

Supplemental Figure 2. Correlation between neuroantigen-specific T cell reactivity and clinical variables. Correlation between age, time from diagnosis, LED, UPDRS part III, and T cell reactivity against MoCA and PINK1, Parkin, OGDH, GBA, SOD1, and LRRK2. T cell reactivity is the sum of the total cytokine response (IFNγ, IL-5, and IL-10) against the respective peptide pools as SFC per 10^6 cultured PBMC. Correlation is indicated by Spearman r and associated p value. Each graph indicates the number of PD patients with the specific clinical variable and T cell reactivity measurement.

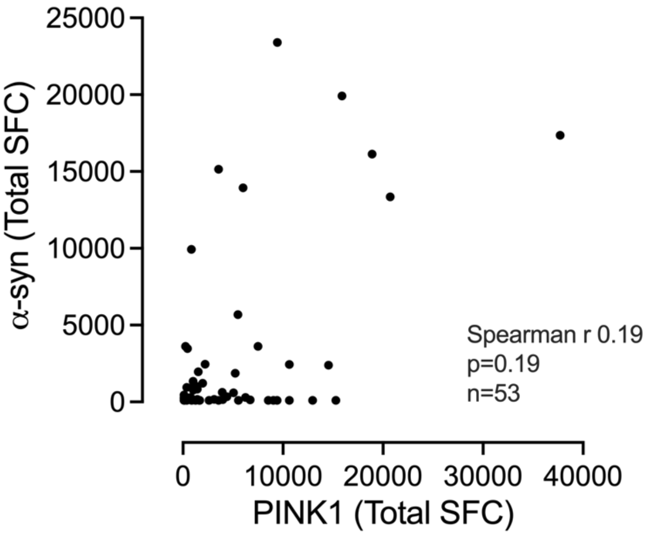

Supplemental Figure 3. Correlation between PINK1- and α-syn-specific T cell reactivity. Correlation between T cell reactivity against PINK1 and α-syn (n=53 patients with PD) responding to either protein. T cell reactivity is the sum of the total cytokine response (IFNγ, IL-5, and IL-10) against the respective peptide pools as SFC per 10^6 cultured PBMC. Correlation is indicated by Spearman r and associated p value.

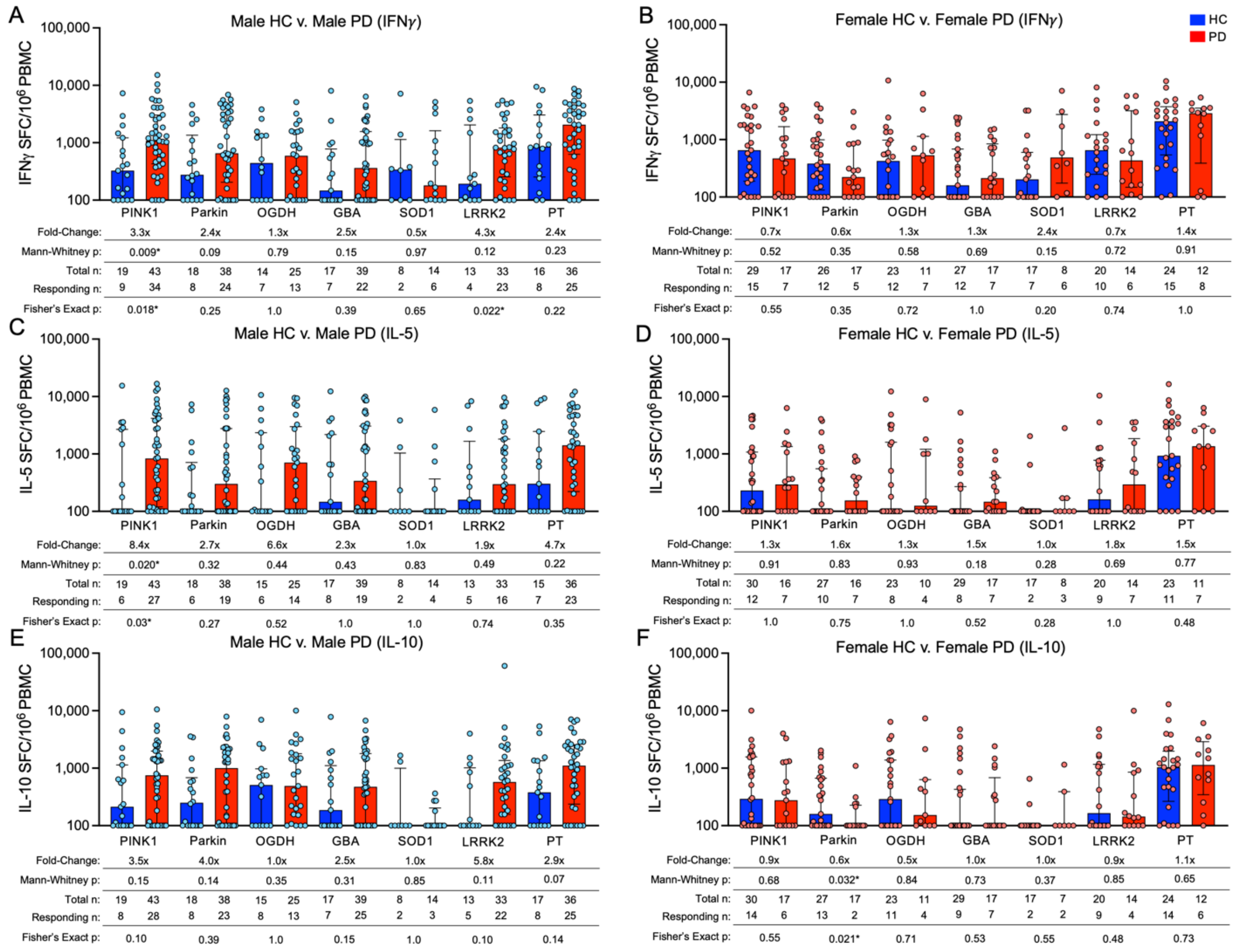

Supplemental Figure 4. Individual cytokine responses among male and female PD patients. Magnitude of the individual cytokine response for a,b) IFNγ c,d) IL-5 e,f) IL-10 among PD and age-matched HC male and female PBMCs. HC (blue bars) and PD (red bars), each circle representing an individual participant. Median ± interquartile range displayed. Fold-change is in comparison to HC response. Two-tailed Mann-Whitney tests were performed between HC and PD antigen-cytokine values. Two-tailed Fisher tests were performed using the geometric mean of the HC group for each individual antigen as a cutoff for the test.

Supplemental Table 1: Cohort characteristics

	HC	PD	
	**n = 49**	**n = 60**	***p* value[a]**
**Sex (male/female)**			
Male	19	43	
Female	30	17	0.0006
**Age in years (mean, SD):**			
Total	65 ± 9	68 ± 8	0.08
Male	67 ± 11	68 ± 9	0.61
Female	64 ± 8	68 ± 6	0.15
**Caucasian (%)[b]:**	87 (34/39)	95 (37/39)	
**Time (years) since PD Diagnosis (mean, range; n):**			
Total	N/A	6 (0-16); 55	
Male	N/A	5.8 (0-16); 41	
Female	N/A	6.6 (0-12); 14	
**MDS-UPDRS Part III[c] (mean, SD; n):**			
Total	2.2 ± 2.8, 17	19.8 ± 8.4; 39	<0.0001
Male	4.7 ± 2.6, 7	20.7 ± 9.0; 29	<0.0001
Female	0.5 ± 1.0, 10	17.3 ± 5.7; 10	<0.0001
**MoCA Score[c] (mean, SD; n):**			
Total	26.3 ± 3.1, 19	26.6 ± 2.7; 49	0.89
Male	26.3 ± 2.7, 7	26.5 ± 2.6; 36	0.79
Female	26.3 ± 3.5, 12	26.8 ± 2.9; 13	0.84
**LED (mean, SD; n):**			
Total	N/A	529 ± 433; 44	
Male	N/A	547 ± 454; 32	
Female	N/A	481 ± 386; 12	

[a] Two-tailed Chi-square test comparing the number of male vs. female participants in each cohort, two-tailed Mann-Whitney test comparing age, MoCA, and MDS-UPDRS (III) between cohorts
[b] Ethnicity was not collected for PPMI participants
[c] MDS-UPDRS (III) & MoCA collected at CUMC, UCSD, and PPMI, few data points for HC.
MDS-UPDRS; Movement Disorder Society- Unified Parkinson’s Disease Rating Scale
MoCA; Montreal Cognitive Assessment
LED; Levodopa equivalent dose

Supplemental Table 2. Peptides and proteins included in the study.

Protein	Protein length	Uniprot reference	Peptide start position	Peptide	Predicted median percentile score	Phosphoserine position	PINK1 individual epitope
PINK1	581	Q9BXM7	1	MAVRQALGRGLQLGR	-		
PINK1	581	Q9BXM7	6	ALGRGLQLGRALLLR	-		
PINK1	581	Q9BXM7	11	LQLGRALLLRFTGKP	-		Yes
PINK1	581	Q9BXM7	16	ALLLRFTGKPGRAYG	-		Yes
PINK1	581	Q9BXM7	21	FTGKPGRAYGLGRPG	-		
PINK1	581	Q9BXM7	26	GRAYGLGRPGPAAGC	-		
PINK1	581	Q9BXM7	31	LGRPGPAAGCVRGER	-		
PINK1	581	Q9BXM7	36	PAAGCVRGERPGWAA	-		
PINK1	581	Q9BXM7	41	VRGERPGWAAGPGAE	-		
PINK1	581	Q9BXM7	46	PGWAAGPGAEPRRVG	-		
PINK1	581	Q9BXM7	51	GPGAEPRRVGLGLPN	-		
PINK1	581	Q9BXM7	56	PRRVGLGLPNRLRFF	-		
PINK1	581	Q9BXM7	61	LGLPNRLRFFRQSVA	-		
PINK1	581	Q9BXM7	66	RLRFFRQSVAGLAAR	-		Yes
PINK1	581	Q9BXM7	71	RQSVAGLAARLQRQF	-		Yes
PINK1	581	Q9BXM7	76	GLAARLQRQFVVRAW	-		
PINK1	581	Q9BXM7	81	LQRQFVVRAWGCAGP	-		
PINK1	581	Q9BXM7	86	VVRAWGCAGPCGRAV	-		
PINK1	581	Q9BXM7	91	GCAGPCGRAVFLAFG	-		
PINK1	581	Q9BXM7	96	CGRAVFLAFGLGLGL	-		
PINK1	581	Q9BXM7	101	FLAFGLGLGLIEEKQ	-		
PINK1	581	Q9BXM7	106	LGLGLIEEKQAESRR	-		
PINK1	581	Q9BXM7	111	IEEKQAESRRAVSAC	-		
PINK1	581	Q9BXM7	116	AESRRAVSACQEIQA	-		
PINK1	581	Q9BXM7	121	AVSACQEIQAIFTQK	-		
PINK1	581	Q9BXM7	126	QEIQAIFTQKSKPGP	-		
PINK1	581	Q9BXM7	131	IFTQKSKPGPDPLDT	-		
PINK1	581	Q9BXM7	136	SKPGPDPLDTRRLQG	-		
PINK1	581	Q9BXM7	141	DPLDTRRLQGFRLEE	-		Yes
PINK1	581	Q9BXM7	146	RRLQGFRLEEYLIGQ	-		Yes
PINK1	581	Q9BXM7	151	FRLEEYLIGQSIGKG	-		Yes
PINK1	581	Q9BXM7	156	YLIGQSIGKGCSAAV	-		
PINK1	581	Q9BXM7	161	SIGKGCSAAVYEATM	-		
PINK1	581	Q9BXM7	166	CSAAVYEATMPTLPQ	-		
PINK1	581	Q9BXM7	171	YEATMPTLPQNLEVT	-		
PINK1	581	Q9BXM7	176	PTLPQNLEVTKSTGL	-		
PINK1	581	Q9BXM7	181	NLEVTKSTGLLPGRG	-		
PINK1	581	Q9BXM7	186	KSTGLLPGRGPGTSA	-		
PINK1	581	Q9BXM7	191	LPGRGPGTSAPGEGQ	-		
PINK1	581	Q9BXM7	196	PGTSAPGEGQERAPG	-		
PINK1	581	Q9BXM7	201	PGEGQERAPGAPAFP	-		
PINK1	581	Q9BXM7	206	ERAPGAPAFPLAIKM	-		
PINK1	581	Q9BXM7	211	APAFPLAIKMMWNIS	-		Yes
PINK1	581	Q9BXM7	216	LAIKMMWNISAGSSS	-		Yes
PINK1	581	Q9BXM7	216	LAIKMMWNISAGXSS		X=phosphoserine aa228	Yes
PINK1	581	Q9BXM7	221	MWNISAGSSSEAILN	-		Yes
PINK1	581	Q9BXM7	226	AGSSSEAILNTMSQE	-		
PINK1	581	Q9BXM7	231	EAILNTMSQELVPAS	-		Yes
PINK1	581	Q9BXM7	236	TMSQELVPASRVALA	-		
PINK1	581	Q9BXM7	241	LVPASRVALAGEYGA	-		
PINK1	581	Q9BXM7	246	RVALAGEYGAVTYRK	-		
PINK1	581	Q9BXM7	251	GEYGAVTYRKSKRGP	-		Yes
PINK1	581	Q9BXM7	256	VTYRKSKRGPKQLAP	-		
PINK1	581	Q9BXM7	261	SKRGPKQLAPHPNII	-		
PINK1	581	Q9BXM7	266	KQLAPHPNIIRVLRA	-		Yes
PINK1	581	Q9BXM7	271	HPNIIRVLRAFTSSV	-		Yes
PINK1	581	Q9BXM7	276	RVLRAFTSSVPLLPG	-		Yes
PINK1	581	Q9BXM7	281	FTSSVPLLPGALVDY	-		

PINK1	581	Q9BXM7	286	PLLPGALVDYPDVLP	-		Yes
PINK1	581	Q9BXM7	291	ALVDYPDVLPSRLHP	-		
PINK1	581	Q9BXM7	296	PDVLPSRLHPEGLGH	-		
PINK1	581	Q9BXM7	301	SRLHPEGLGHGRTLF	-		
PINK1	581	Q9BXM7	306	EGLGHGRTLFLVMKN	-		
PINK1	581	Q9BXM7	311	GRTLFLVMKNYPCTL	-		
PINK1	581	Q9BXM7	316	LVMKNYPCTLRQYLC	-		
PINK1	581	Q9BXM7	321	YPCTLRQYLCVNTPS	-		
PINK1	581	Q9BXM7	326	RQYLCVNTPSPRLAA	-		
PINK1	581	Q9BXM7	331	VNTPSPRLAAMMLLQ	-		
PINK1	581	Q9BXM7	336	PRLAAMMLLQLLEGV	-		Yes
PINK1	581	Q9BXM7	341	MMLLQLLEGVDHLVQ	-		Yes
PINK1	581	Q9BXM7	346	LLEGVDHLVQQGIAH	-		
PINK1	581	Q9BXM7	351	DHLVQQGIAHRDLKS	-		
PINK1	581	Q9BXM7	356	QGIAHRDLKSDNILV	-		Yes
PINK1	581	Q9BXM7	361	RDLKSDNILVELDPD	-		Yes
PINK1	581	Q9BXM7	366	DNILVELDPDGCPWL	-		
PINK1	581	Q9BXM7	371	ELDPDGCPWLVIADF	-		Yes
PINK1	581	Q9BXM7	376	GCPWLVIADFGCCLA	-		Yes
PINK1	581	Q9BXM7	381	VIADFGCCLADESIG	-		
PINK1	581	Q9BXM7	386	GCCLADESIGLQLPF	-		
PINK1	581	Q9BXM7	391	DESIGLQLPFSSWYV	-		
PINK1	581	Q9BXM7	391	DESIGLQLPFSXWYV	-	X=phosphoserine aa402	Yes
PINK1	581	Q9BXM7	396	LQLPFSSWYVDRGGN	-		
PINK1	581	Q9BXM7	401	SSWYVDRGGNGCLMA	-		
PINK1	581	Q9BXM7	406	DRGGNGCLMAPEVST	-		
PINK1	581	Q9BXM7	411	GCLMAPEVSTARPGP	-		
PINK1	581	Q9BXM7	416	PEVSTARPGPRAVID	-		
PINK1	581	Q9BXM7	421	ARPGPRAVIDYSKAD	-		
PINK1	581	Q9BXM7	426	RAVIDYSKADAWAVG	-		
PINK1	581	Q9BXM7	431	YSKADAWAVGAIAYE	-		
PINK1	581	Q9BXM7	436	AWAVGAIAYEIFGLV	-		
PINK1	581	Q9BXM7	441	AIAYEIFGLVNPFYG	-		
PINK1	581	Q9BXM7	446	IFGLVNPFYGQGKAH	-		
PINK1	581	Q9BXM7	451	NPFYGQGKAHLESRS	-		
PINK1	581	Q9BXM7	456	QGKAHLESRSYQEAQ	-		
PINK1	581	Q9BXM7	461	LESRSYQEAQLPALP	-		
PINK1	581	Q9BXM7	466	YQEAQLPALPESVPP	-		
PINK1	581	Q9BXM7	471	LPALPESVPPDVRQL	-		
PINK1	581	Q9BXM7	476	ESVPPDVRQLVRALL	-		Yes
PINK1	581	Q9BXM7	481	DVRQLVRALLQREAS	-		Yes
PINK1	581	Q9BXM7	486	VRALLQREASKRPSA	-		
PINK1	581	Q9BXM7	491	QREASKRPSARVAAN	-		
PINK1	581	Q9BXM7	496	KRPSARVAANVLHLS	-		Yes
PINK1	581	Q9BXM7	501	RVAANVLHLSLWGEH	-		
PINK1	581	Q9BXM7	506	VLHLSLWGEHILALK	-		Yes
PINK1	581	Q9BXM7	511	LWGEHILALKNLKLD	-		Yes
PINK1	581	Q9BXM7	516	ILALKNLKLDKMVGW	-		Yes
PINK1	581	Q9BXM7	521	NLKLDKMVGWLLQQS	-		Yes
PINK1	581	Q9BXM7	526	KMVGWLLQQSAATLL	-		
PINK1	581	Q9BXM7	531	LLQQSAATLLANRLT	-		Yes
PINK1	581	Q9BXM7	536	AATLLANRLTEKCCV	-		Yes
PINK1	581	Q9BXM7	541	ANRLTEKCCVETKMK	-		
PINK1	581	Q9BXM7	546	EKCCVETKMKMLFLA	-		
PINK1	581	Q9BXM7	551	ETKMKMLFLANLECE	-		
PINK1	581	Q9BXM7	556	MLFLANLECETLCQA	-		
PINK1	581	Q9BXM7	561	NLECETLCQAALLLC	-		
PINK1	581	Q9BXM7	566	TLCQAALLLCSWRAA	-		
PINK1	581	Q9BXM7	567	LCQAALLLCSWRAAL	-		Yes
PARKIN	465	O60260	1	MIVFVRFNSSHGFPV	-		
PARKIN	465	O60260	6	RFNSSHGFPVEVDSD	-		
PARKIN	465	O60260	11	HGFPVEVDSDTSIFQ	-		
PARKIN	465	O60260	16	EVDSDTSIFQLKEVV	-		

PARKIN	465	O60260	21	TSIFQLKEVVAKRQG	-		
PARKIN	465	O60260	26	LKEVVAKRQGVPADQ	-		
PARKIN	465	O60260	31	AKRQGVPADQLRVIF	-		
PARKIN	465	O60260	36	VPADQLRVIFAGKEL	-		
PARKIN	465	O60260	41	LRVIFAGKELRNDWT	-		
PARKIN	465	O60260	46	AGKELRNDWTVQNCD	-		
PARKIN	465	O60260	51	RNDWTVQNCDLDQQS	-		
PARKIN	465	O60260	51	RNDWTVQNCDLDQQX	-	X=phosphoserine aa65	
PARKIN	465	O60260	56	VQNCDLDQQSIVHIV	-		
PARKIN	465	O60260	56	VQNCDLDQQXIVHIV	-	X=phosphoserine aa65	
PARKIN	465	O60260	61	LDQQSIVHIVQRPWR	-		
PARKIN	465	O60260	61	LDQQXIVHIVQRPWR	-	X=phosphoserine aa65	
PARKIN	465	O60260	66	IVHIVQRPWRKGQEM	-		
PARKIN	465	O60260	71	QRPWRKGQEMNATGG	-		
PARKIN	465	O60260	76	KGQEMNATGGDDPRN	-		
PARKIN	465	O60260	81	NATGGDDPRNAAGGC	-		
PARKIN	465	O60260	86	DDPRNAAGGCEREPQ	-		
PARKIN	465	O60260	91	AAGGCEREPQSLTRV	-		
PARKIN	465	O60260	96	EREPQSLTRVDLSSS	-		
PARKIN	465	O60260	101	SLTRVDLSSSVLPGD	-		
PARKIN	465	O60260	106	DLSSSVLPGDSVGLA	-		
PARKIN	465	O60260	111	VLPGDSVGLAVILHT	-		
PARKIN	465	O60260	116	SVGLAVILHTDSRKD	-		
PARKIN	465	O60260	121	VILHTDSRKDSPPAG	-		
PARKIN	465	O60260	126	DSRKDSPPAGSPAGR	-		
PARKIN	465	O60260	131	SPPAGSPAGRSIYNS	-		
PARKIN	465	O60260	136	SPAGRSIYNSFYVYC	-		
PARKIN	465	O60260	141	SIYNSFYVYCKGPCQ	-		
PARKIN	465	O60260	146	FYVYCKGPCQRVQPG	-		
PARKIN	465	O60260	151	KGPCQRVQPGKLRVQ	-		
PARKIN	465	O60260	156	RVQPGKLRVQCSTCR	-		
PARKIN	465	O60260	161	KLRVQCSTCRQATLT	-		
PARKIN	465	O60260	166	CSTCRQATLTLTQGP	-		
PARKIN	465	O60260	171	QATLTLTQGPSCWDD	-		
PARKIN	465	O60260	176	LTQGPSCWDDVLIPN	-		
PARKIN	465	O60260	181	SCWDDVLIPNRMSGE	-		
PARKIN	465	O60260	186	VLIPNRMSGECQSPH	-		
PARKIN	465	O60260	191	RMSGECQSPHCPGTS	-		
PARKIN	465	O60260	196	CQSPHCPGTSAEFFF	-		
PARKIN	465	O60260	201	CPGTSAEFFFKCGAH	-		
PARKIN	465	O60260	206	AEFFFKCGAHPTSDK	-		
PARKIN	465	O60260	211	KCGAHPTSDKETSVA	-		
PARKIN	465	O60260	216	PTSDKETSVALHLIA	-		
PARKIN	465	O60260	221	ETSVALHLIATNSRN	-		
PARKIN	465	O60260	226	LHLIATNSRNITCIT	-		
PARKIN	465	O60260	231	TNSRNITCITCTDVR	-		
PARKIN	465	O60260	236	ITCITCTDVRSPVLV	-		
PARKIN	465	O60260	241	CTDVRSPVLVFQCNS	-		
PARKIN	465	O60260	246	SPVLVFQCNSRHVIC	-		
PARKIN	465	O60260	251	FQCNSRHVICLDCFH	-		
PARKIN	465	O60260	256	RHVICLDCFHLYCVT	-		
PARKIN	465	O60260	261	LDCFHLYCVTRLNDR	-		
PARKIN	465	O60260	266	LYCVTRLNDRQFVHD	-		
PARKIN	465	O60260	271	RLNDRQFVHDPQLGY	-		
PARKIN	465	O60260	276	QFVHDPQLGYSLPCV	-		
PARKIN	465	O60260	281	PQLGYSLPCVAGCPN	-		
PARKIN	465	O60260	286	SLPCVAGCPNSLIKE	-		
PARKIN	465	O60260	291	AGCPNSLIKELHHFR	-		
PARKIN	465	O60260	296	SLIKELHHFRILGEE	-		
PARKIN	465	O60260	301	LHHFRILGEEQYNRY	-		

PARKIN	465	O60260	306	ILGEEQYNRYQQYGA	-		
PARKIN	465	O60260	311	QYNRYQQYGAEECVL	-		
PARKIN	465	O60260	316	QQYGAEECVLQMGGV	-		
PARKIN	465	O60260	321	EECVLQMGGVLCPRP	-		
PARKIN	465	O60260	326	QMGGVLCPRPGCGAG	-		
PARKIN	465	O60260	331	LCPRPGCGAGLLPEP	-		
PARKIN	465	O60260	336	GCGAGLLPEPDQRKV	-		
PARKIN	465	O60260	341	LLPEPDQRKVTCEGG	-		
PARKIN	465	O60260	346	DQRKVTCEGGNGLGC	-		
PARKIN	465	O60260	351	TCEGGNGLGCGFAFC	-		
PARKIN	465	O60260	356	NGLGCGFAFCRECKE	-		
PARKIN	465	O60260	361	GFAFCRECKEAYHEG	-		
PARKIN	465	O60260	366	RECKEAYHEGECSAV	-		
PARKIN	465	O60260	371	AYHEGECSAVFEASG	-		
PARKIN	465	O60260	376	ECSAVFEASGTTTQA	-		
PARKIN	465	O60260	381	FEASGTTTQAYRVDE	-		
PARKIN	465	O60260	386	TTTQAYRVDERAAEQ	-		
PARKIN	465	O60260	391	YRVDERAAEQARWEA	-		
PARKIN	465	O60260	396	RAAEQARWEAASKET	-		
PARKIN	465	O60260	401	ARWEAASKETIKKTT	-		
PARKIN	465	O60260	406	ASKETIKKTTKPCPR	-		
PARKIN	465	O60260	411	IKKTTKPCPRCHVPV	-		
PARKIN	465	O60260	416	KPCPRCHVPVEKNGG	-		
PARKIN	465	O60260	421	CHVPVEKNGGCMHMK	-		
PARKIN	465	O60260	426	EKNGGCMHMKCPQPQ	-		
PARKIN	465	O60260	431	CMHMKCPQPQCRLEW	-		
PARKIN	465	O60260	436	CPQPQCRLEWCWNCG	-		
PARKIN	465	O60260	441	CRLEWCWNCGCEWNR	-		
PARKIN	465	O60260	446	CWNCGCEWNRVCMGD	-		
PARKIN	465	O60260	451	CEWNRVCMGDHWFDV	-		
OGDH	1023	Q02218	1	MFHLRTCAAKLRPLT	-		
OGDH	1023	Q02218	6	TCAAKLRPLTASQTV	-		
OGDH	1023	Q02218	11	LRPLTASQTVKTFSQ	-		
OGDH	1023	Q02218	16	ASQTVKTFSQNRPAA	-		
OGDH	1023	Q02218	21	KTFSQNRPAAARTFQ	-		
OGDH	1023	Q02218	26	NRPAAARTFQQIRCY	-		
OGDH	1023	Q02218	31	ARTFQQIRCYSAPVA	-		
OGDH	1023	Q02218	36	QIRCYSAPVAAEPFL	-		
OGDH	1023	Q02218	41	SAPVAAEPFLSGTSS	-		
OGDH	1023	Q02218	46	AEPFLSGTSSNYVEE	-		
OGDH	1023	Q02218	51	SGTSSNYVEEMYCAW	-		
OGDH	1023	Q02218	56	NYVEEMYCAWLENPK	-		
OGDH	1023	Q02218	61	MYCAWLENPKSVHKS	-		
OGDH	1023	Q02218	66	LENPKSVHKSWDIFF	-		
OGDH	1023	Q02218	71	SVHKSWDIFFRNTNA	-		
OGDH	1023	Q02218	76	WDIFFRNTNAGAPPG	-		
OGDH	1023	Q02218	81	RNTNAGAPPGTAYQS	-		
OGDH	1023	Q02218	86	GAPPGTAYQSPLPLS	-		
OGDH	1023	Q02218	91	TAYQSPLPLSRGSLA	-		
OGDH	1023	Q02218	96	PLPLSRGSLAAVAHA	-		
OGDH	1023	Q02218	101	RGSLAAVAHAQSLVE	-		
OGDH	1023	Q02218	106	AVAHAQSLVEAQPNV	-		
OGDH	1023	Q02218	111	QSLVEAQPNVDKLVE	-		
OGDH	1023	Q02218	116	AQPNVDKLVEDHLAV	-		
OGDH	1023	Q02218	121	DKLVEDHLAVQSLIR	-		
OGDH	1023	Q02218	126	DHLAVQSLIRAYQIR	-		
OGDH	1023	Q02218	131	QSLIRAYQIRGHHVA	-		
OGDH	1023	Q02218	136	AYQIRGHHVAQLDPL	-		
OGDH	1023	Q02218	141	GHHVAQLDPLGILDA	-		
OGDH	1023	Q02218	146	QLDPLGILDADLDSS	-		
OGDH	1023	Q02218	151	GILDADLDSSVPADI	-		
OGDH	1023	Q02218	156	DLDSSVPADIISSTD	-		
OGDH	1023	Q02218	161	VPADIISSTDKLGFY	-		

OGDH	1023	Q02218	166	ISSTDKLGFYGLDES	-		
OGDH	1023	Q02218	171	KLGFYGLDESDLDKV	-		
OGDH	1023	Q02218	176	GLDESDLDKVFHLPT	-		
OGDH	1023	Q02218	181	DLDKVFHLPTTTFIG	-		
OGDH	1023	Q02218	186	FHLPTTTFIGGQESA	-		
OGDH	1023	Q02218	191	TTFIGGQESALPLRE	-		
OGDH	1023	Q02218	196	GQESALPLREIIRRL	-		
OGDH	1023	Q02218	201	LPLREIIRRLEMAYC	-		
OGDH	1023	Q02218	206	IIRRLEMAYCQHIGV	-		
OGDH	1023	Q02218	211	EMAYCQHIGVEFMFI	-		
OGDH	1023	Q02218	216	QHIGVEFMFINDLEQ	-		
OGDH	1023	Q02218	221	EFMFINDLEQCQWIR	-		
OGDH	1023	Q02218	226	NDLEQCQWIRQKFET	-		
OGDH	1023	Q02218	231	CQWIRQKFETPGIMQ	-		
OGDH	1023	Q02218	236	QKFETPGIMQFTNEE	-		
OGDH	1023	Q02218	241	PGIMQFTNEEKRTLL	-		
OGDH	1023	Q02218	246	FTNEEKRTLLARLVR	-		
OGDH	1023	Q02218	251	KRTLLARLVRSTRFE	-		
OGDH	1023	Q02218	256	ARLVRSTRFEEFLQR	-		
OGDH	1023	Q02218	261	STRFEEFLQRKWSSE	-		
OGDH	1023	Q02218	266	EFLQRKWSSEKRFGL	-		
OGDH	1023	Q02218	271	KWSSEKRFGLEGCEV	-		
OGDH	1023	Q02218	276	KRFGLEGCEVLIPAL	-		
OGDH	1023	Q02218	281	EGCEVLIPALKTIID	-		
OGDH	1023	Q02218	286	LIPALKTIIDKSSEN	-		
OGDH	1023	Q02218	291	KTIIDKSSENGVDYV	-		
OGDH	1023	Q02218	296	KSSENGVDYVIMGMP	-		
OGDH	1023	Q02218	301	GVDYVIMGMPHRGRL	-		
OGDH	1023	Q02218	306	IMGMPHRGRLNVLAN	-		
OGDH	1023	Q02218	311	HRGRLNVLANVIRKE	-		
OGDH	1023	Q02218	316	NVLANVIRKELEQIF	-		
OGDH	1023	Q02218	321	VIRKELEQIFCQFDS	-		
OGDH	1023	Q02218	326	LEQIFCQFDSKLEAA	-		
OGDH	1023	Q02218	331	CQFDSKLEAADEGSG	-		
OGDH	1023	Q02218	336	KLEAADEGSGDVKYH	-		
OGDH	1023	Q02218	341	DEGSGDVKYHLGMYH	-		
OGDH	1023	Q02218	346	DVKYHLGMYHRRINR	-		
OGDH	1023	Q02218	351	LGMYHRRINRVTDRN	-		
OGDH	1023	Q02218	356	RRINRVTDRNITLSL	-		
OGDH	1023	Q02218	361	VTDRNITLSLVANPS	-		
OGDH	1023	Q02218	366	ITLSLVANPSHLEAA	-		
OGDH	1023	Q02218	371	VANPSHLEAADPVVM	-		
OGDH	1023	Q02218	376	HLEAADPVVMGKTKA	-		
OGDH	1023	Q02218	381	DPVVMGKTKAEQFYC	-		
OGDH	1023	Q02218	386	GKTKAEQFYCGDTEG	-		
OGDH	1023	Q02218	391	EQFYCGDTEGKKVMS	-		
OGDH	1023	Q02218	396	GDTEGKKVMSILLHG	-		
OGDH	1023	Q02218	401	KKVMSILLHGDAAFA	-		
OGDH	1023	Q02218	406	ILLHGDAAFAGQGIV	-		
OGDH	1023	Q02218	411	DAAFAGQGIVYETFH	-		
OGDH	1023	Q02218	416	GQGIVYETFHLSDLP	-		
OGDH	1023	Q02218	421	YETFHLSDLPSYTTH	-		
OGDH	1023	Q02218	426	LSDLPSYTTHGTVHV	-		
OGDH	1023	Q02218	431	SYTTHGTVHVVVNNQ	-		
OGDH	1023	Q02218	436	GTVHVVVNNQIGFTT	-		
OGDH	1023	Q02218	441	VVNNQIGFTTDPRMA	-		
OGDH	1023	Q02218	446	IGFTTDPRMARSSPY	-		
OGDH	1023	Q02218	451	DPRMARSSPYPTDVA	-		
OGDH	1023	Q02218	456	RSSPYPTDVARVVNA	-		
OGDH	1023	Q02218	461	PTDVARVVNAPIFHV	-		
OGDH	1023	Q02218	466	RVVNAPIFHVNSDDP	-		
OGDH	1023	Q02218	471	PIFHVNSDDPEAVMY	-		
OGDH	1023	Q02218	476	NSDDPEAVMYVCKVA	-		

OGDH	1023	Q02218	481	EAVMYVCKVAAEWRS	-		
OGDH	1023	Q02218	486	VCKVAAEWRSTFHKD	-		
OGDH	1023	Q02218	491	AEWRSTFHKDVVVDL	-		
OGDH	1023	Q02218	496	TFHKDVVVDLVCYRR	-		
OGDH	1023	Q02218	501	VVVDLVCYRRNGHNE	-		
OGDH	1023	Q02218	506	VCYRRNGHNEMDEPM	-		
OGDH	1023	Q02218	511	NGHNEMDEPMFTQPL	-		
OGDH	1023	Q02218	516	MDEPMFTQPLMYKQI	-		
OGDH	1023	Q02218	521	FTQPLMYKQIRKQKP	-		
OGDH	1023	Q02218	526	MYKQIRKQKPVLQKY	-		
OGDH	1023	Q02218	531	RKQKPVLQKYAELLV	-		
OGDH	1023	Q02218	536	VLQKYAELLVSQGVV	-		
OGDH	1023	Q02218	541	AELLVSQGVVNQPEY	-		
OGDH	1023	Q02218	546	SQGVVNQPEYEEEIS	-		
OGDH	1023	Q02218	551	NQPEYEEEISKYDKI	-		
OGDH	1023	Q02218	556	EEEISKYDKICEEAF	-		
OGDH	1023	Q02218	561	KYDKICEEAFARSKD	-		
OGDH	1023	Q02218	566	CEEAFARSKDEKILH	-		
OGDH	1023	Q02218	571	ARSKDEKILHIKHWL	-		
OGDH	1023	Q02218	576	EKILHIKHWLDSPWP	-		
OGDH	1023	Q02218	581	IKHWLDSPWPGFFTL	-		
OGDH	1023	Q02218	586	DSPWPGFFTLDGQPR	-		
OGDH	1023	Q02218	591	GFFTLDGQPRSMSCP	-		
OGDH	1023	Q02218	596	DGQPRSMSCPSTGLT	-		
OGDH	1023	Q02218	601	SMSCPSTGLTEDILT	-		
OGDH	1023	Q02218	606	STGLTEDILTHIGNV	-		
OGDH	1023	Q02218	611	EDILTHIGNVASSVP	-		
OGDH	1023	Q02218	616	HIGNVASSVPVENFT	-		
OGDH	1023	Q02218	621	ASSVPVENFTIHGGL	-		
OGDH	1023	Q02218	626	VENFTIHGGLSRILK	-		
OGDH	1023	Q02218	631	IHGGLSRILKTRGEM	-		
OGDH	1023	Q02218	636	SRILKTRGEMVKNRT	-		
OGDH	1023	Q02218	641	TRGEMVKNRTVDWAL	-		
OGDH	1023	Q02218	646	VKNRTVDWALAEYMA	-		
OGDH	1023	Q02218	651	VDWALAEYMAFGSLL	-		
OGDH	1023	Q02218	656	AEYMAFGSLLKEGIH	-		
OGDH	1023	Q02218	661	FGSLLKEGIHIRLSG	-		
OGDH	1023	Q02218	666	KEGIHIRLSGQDVER	-		
OGDH	1023	Q02218	671	IRLSGQDVERGTFSH	-		
OGDH	1023	Q02218	676	QDVERGTFSHRHHVL	-		
OGDH	1023	Q02218	681	GTFSHRHHVLHDQNV	-		
OGDH	1023	Q02218	686	RHHVLHDQNVDKRTC	-		
OGDH	1023	Q02218	691	HDQNVDKRTCIPMNH	-		
OGDH	1023	Q02218	696	DKRTCIPMNHLWPNQ	-		
OGDH	1023	Q02218	701	IPMNHLWPNQAPYTV	-		
OGDH	1023	Q02218	706	LWPNQAPYTVCNSSL	-		
OGDH	1023	Q02218	711	APYTVCNSSLSEYGV	-		
OGDH	1023	Q02218	716	CNSSLSEYGVLGFEL	-		
OGDH	1023	Q02218	721	SEYGVLGFELGFAMA	-		
OGDH	1023	Q02218	726	LGFELGFAMASPNAL	-		
OGDH	1023	Q02218	731	GFAMASPNALVLWEA	-		
OGDH	1023	Q02218	736	SPNALVLWEAQFGDF	-		
OGDH	1023	Q02218	741	VLWEAQFGDFHNTAQ	-		
OGDH	1023	Q02218	746	QFGDFHNTAQCIIDQ	-		
OGDH	1023	Q02218	751	HNTAQCIIDQFICPG	-		
OGDH	1023	Q02218	756	CIIDQFICPGQAKWV	-		
OGDH	1023	Q02218	761	FICPGQAKWVRQNGI	-		
OGDH	1023	Q02218	766	QAKWVRQNGIVLLLP	-		
OGDH	1023	Q02218	771	RQNGIVLLLPHGMEG	-		
OGDH	1023	Q02218	776	VLLLPHGMEGMGPEH	-		
OGDH	1023	Q02218	781	HGMEGMGPEHSSARP	-		
OGDH	1023	Q02218	786	MGPEHSSARPERFLQ	-		
OGDH	1023	Q02218	791	SSARPERFLQMCNDD	-		

OGDH	1023	Q02218	796	ERFLQMCNDDPDVLP	-		
OGDH	1023	Q02218	801	MCNDDPDVLPDLKEA	-		
OGDH	1023	Q02218	806	PDVLPDLKEANFDIN	-		
OGDH	1023	Q02218	811	DLKEANFDINQLYDC	-		
OGDH	1023	Q02218	816	NFDINQLYDCNWVVV	-		
OGDH	1023	Q02218	821	QLYDCNWVVVNCSTP	-		
OGDH	1023	Q02218	826	NWVVVNCSTPGNFFH	-		
OGDH	1023	Q02218	831	NCSTPGNFFHVLRRQ	-		
OGDH	1023	Q02218	836	GNFFHVLRRQILLPF	-		
OGDH	1023	Q02218	841	VLRRQILLPFRKPLI	-		
OGDH	1023	Q02218	846	ILLPFRKPLIIFTPK	-		
OGDH	1023	Q02218	851	RKPLIIFTPKSLLRH	-		
OGDH	1023	Q02218	856	IFTPKSLLRHPEARS	-		
OGDH	1023	Q02218	861	SLLRHPEARSSFDEM	-		
OGDH	1023	Q02218	866	PEARSSFDEMLPGTH	-		
OGDH	1023	Q02218	871	SFDEMLPGTHFQRVI	-		
OGDH	1023	Q02218	876	LPGTHFQRVIPEDGP	-		
OGDH	1023	Q02218	881	FQRVIPEDGPAAQNP	-		
OGDH	1023	Q02218	886	PEDGPAAQNPENVKR	-		
OGDH	1023	Q02218	891	AAQNPENVKRLLFCT	-		
OGDH	1023	Q02218	896	ENVKRLLFCTGKVYY	-		
OGDH	1023	Q02218	901	LLFCTGKVYYDLTRE	-		
OGDH	1023	Q02218	906	GKVYYDLTRERKARD	-		
OGDH	1023	Q02218	911	DLTRERKARDMVGQV	-		
OGDH	1023	Q02218	916	RKARDMVGQVAITRI	-		
OGDH	1023	Q02218	921	MVGQVAITRIEQLSP	-		
OGDH	1023	Q02218	926	AITRIEQLSPFPFDL	-		
OGDH	1023	Q02218	931	EQLSPFPFDLLLKEV	-		
OGDH	1023	Q02218	936	FPFDLLLKEVQKYPN	-		
OGDH	1023	Q02218	941	LLKEVQKYPNAELAW	-		
OGDH	1023	Q02218	946	QKYPNAELAWCQEEH	-		
OGDH	1023	Q02218	951	AELAWCQEEHKNQGY	-		
OGDH	1023	Q02218	956	CQEEHKNQGYYDYVK	-		
OGDH	1023	Q02218	961	KNQGYYDYVKPRLRT	-		
OGDH	1023	Q02218	966	YDYVKPRLRTTISRA	-		
OGDH	1023	Q02218	971	PRLRTTISRAKPVWY	-		
OGDH	1023	Q02218	976	TISRAKPVWYAGRDP	-		
OGDH	1023	Q02218	981	KPVWYAGRDPAAAPA	-		
OGDH	1023	Q02218	986	AGRDPAAAPATGNKK	-		
OGDH	1023	Q02218	991	AAAPATGNKKTHLTE	-		
OGDH	1023	Q02218	996	TGNKKTHLTELQRLL	-		
OGDH	1023	Q02218	1001	THLTELQRLLDTAFD	-		
OGDH	1023	Q02218	1006	LQRLLDTAFDLDVFK	-		
OGDH	1023	Q02218	1009	LLDTAFDLDVFKNFS	-		
GBA	503	P04062	1	MEFSSPSREECPKPL	-		
GBA	503	P04062	6	PSREECPKPLSRVSI	-		
GBA	503	P04062	11	CPKPLSRVSIMAGSL	-		
GBA	503	P04062	16	SRVSIMAGSLTGLLL	-		
GBA	503	P04062	21	MAGSLTGLLLLQAVS	-		
GBA	503	P04062	26	TGLLLLQAVSWASGA	-		
GBA	503	P04062	31	LQAVSWASGARPCIP	-		
GBA	503	P04062	36	WASGARPCIPKSFGY	-		
GBA	503	P04062	41	RPCIPKSFGYSSVVC	-		
GBA	503	P04062	46	KSFGYSSVVCVCNAT	-		
GBA	503	P04062	51	SSVVCVCNATYCDSF	-		
GBA	503	P04062	56	VCNATYCDSFDPPTF	-		
GBA	503	P04062	61	YCDSFDPPTFPALGT	-		
GBA	503	P04062	66	DPPTFPALGTFSRYE	-		
GBA	503	P04062	71	PALGTFSRYESTRSG	-		
GBA	503	P04062	76	FSRYESTRSGRRMEL	-		
GBA	503	P04062	81	STRSGRRMELSMGPI	-		
GBA	503	P04062	86	RRMELSMGPIQANHT	-		
GBA	503	P04062	91	SMGPIQANHTGTGLL	-		

GBA	503	P04062	96	QANHTGTGLLLTLQP	-		
GBA	503	P04062	101	GTGLLLTLQPEQKFQ	-		
GBA	503	P04062	106	LTLQPEQKFQKVKGF	-		
GBA	503	P04062	111	EQKFQKVKGFGGAMT	-		
GBA	503	P04062	116	KVKGFGGAMTDAAAL	-		
GBA	503	P04062	121	GGAMTDAAALNILAL	-		
GBA	503	P04062	126	DAAALNILALSPPAQ	-		
GBA	503	P04062	131	NILALSPPAQNLLLK	-		
GBA	503	P04062	136	SPPAQNLLLKSYFSE	-		
GBA	503	P04062	141	NLLLKSYFSEEGIGY	-		
GBA	503	P04062	146	SYFSEEGIGYNIIRV	-		
GBA	503	P04062	151	EGIGYNIIRVPMASC	-		
GBA	503	P04062	156	NIIRVPMASCDFSIR	-		
GBA	503	P04062	161	PMASCDFSIRTYTYA	-		
GBA	503	P04062	166	DFSIRTYTYADTPDD	-		
GBA	503	P04062	171	TYTYADTPDDFQLHN	-		
GBA	503	P04062	176	DTPDDFQLHNFSLPE	-		
GBA	503	P04062	181	FQLHNFSLPEEDTKL	-		
GBA	503	P04062	186	FSLPEEDTKLKIPLI	-		
GBA	503	P04062	191	EDTKLKIPLIHRALQ	-		
GBA	503	P04062	196	KIPLIHRALQLAQRP	-		
GBA	503	P04062	201	HRALQLAQRPVSLLA	-		
GBA	503	P04062	206	LAQRPVSLLASPWTS	-		
GBA	503	P04062	211	VSLLASPWTSPTWLK	-		
GBA	503	P04062	216	SPWTSPTWLKTNGAV	-		
GBA	503	P04062	221	PTWLKTNGAVNGKGS	-		
GBA	503	P04062	226	TNGAVNGKGSLKGQP	-		
GBA	503	P04062	231	NGKGSLKGQPGDIYH	-		
GBA	503	P04062	236	LKGQPGDIYHQTWAR	-		
GBA	503	P04062	241	GDIYHQTWARYFVKF	-		
GBA	503	P04062	246	QTWARYFVKFLDAYA	-		
GBA	503	P04062	251	YFVKFLDAYAEHKLQ	-		
GBA	503	P04062	256	LDAYAEHKLQFWAVT	-		
GBA	503	P04062	261	EHKLQFWAVTAENEP	-		
GBA	503	P04062	266	FWAVTAENEPSAGLL	-		
GBA	503	P04062	271	AENEPSAGLLSGYPF	-		
GBA	503	P04062	276	SAGLLSGYPFQCLGF	-		
GBA	503	P04062	281	SGYPFQCLGFTPEHQ	-		
GBA	503	P04062	286	QCLGFTPEHQRDFIA	-		
GBA	503	P04062	291	TPEHQRDFIARDLGP	-		
GBA	503	P04062	296	RDFIARDLGPTLANS	-		
GBA	503	P04062	301	RDLGPTLANSTHHNV	-		
GBA	503	P04062	306	TLANSTHHNVRLLML	-		
GBA	503	P04062	311	THHNVRLLMLDDQRL	-		
GBA	503	P04062	316	RLLMLDDQRLLLPHW	-		
GBA	503	P04062	321	DDQRLLLPHWAKVVL	-		
GBA	503	P04062	326	LLPHWAKVVLTDPEA	-		
GBA	503	P04062	331	AKVVLTDPEAAKYVH	-		
GBA	503	P04062	336	TDPEAAKYVHGIAVH	-		
GBA	503	P04062	341	AKYVHGIAVHWYLDF	-		
GBA	503	P04062	346	GIAVHWYLDFLAPAK	-		
GBA	503	P04062	351	WYLDFLAPAKATLGE	-		
GBA	503	P04062	356	LAPAKATLGETHRLF	-		
GBA	503	P04062	361	ATLGETHRLFPNTML	-		
GBA	503	P04062	366	THRLFPNTMLFASEA	-		
GBA	503	P04062	371	PNTMLFASEACVGSK	-		
GBA	503	P04062	376	FASEACVGSKFWEQS	-		
GBA	503	P04062	381	CVGSKFWEQSVRLGS	-		
GBA	503	P04062	386	FWEQSVRLGSWDRGM	-		
GBA	503	P04062	391	VRLGSWDRGMQYSHS	-		
GBA	503	P04062	396	WDRGMQYSHSIITNL	-		
GBA	503	P04062	401	QYSHSIITNLLYHVV	-		
GBA	503	P04062	406	IITNLLYHVVGWTDW	-		

GBA	503	P04062	411	LYHVVGWTDWNLALN	-		
GBA	503	P04062	416	GWTDWNLALNPEGGP	-		
GBA	503	P04062	421	NLALNPEGGPNWVRN	-		
GBA	503	P04062	426	PEGGPNWVRNFVDSP	-		
GBA	503	P04062	431	NWVRNFVDSPIIVDI	-		
GBA	503	P04062	436	FVDSPIIVDITKDTF	-		
GBA	503	P04062	441	IIVDITKDTFYKQPM	-		
GBA	503	P04062	446	TKDTFYKQPMFYHLG	-		
GBA	503	P04062	451	YKQPMFYHLGHFSKF	-		
GBA	503	P04062	456	FYHLGHFSKFIPEGS	-		
GBA	503	P04062	461	HFSKFIPEGSQRVGL	-		
GBA	503	P04062	466	IPEGSQRVGLVASQK	-		
GBA	503	P04062	471	QRVGLVASQKNDLDA	-		
GBA	503	P04062	476	VASQKNDLDAVALMH	-		
GBA	503	P04062	481	NDLDAVALMHPDGSA	-		
GBA	503	P04062	486	VALMHPDGSAVVVVL	-		
GBA	503	P04062	491	PDGSAVVVVLNRSSK	-		
GBA	503	P04062	496	VVVVLNRSSKDVPLT	-		
GBA	503	P04062	501	NRSSKDVPLTIKDPA	-		
GBA	503	P04062	506	DVPLTIKDPAVGFLE	-		
GBA	503	P04062	511	IKDPAVGFLETISPG	-		
GBA	503	P04062	516	VGFLETISPGYSIHT	-		
GBA	503	P04062	521	TISPGYSIHTYLWRR	-		
GBA	503	P04062	522	ISPGYSIHTYLWRRQ	-		
SOD1	153	P00441	1	MATKAVCVLKGDGPV	-		
SOD1	153	P00441	6	VCVLKGDGPVQGIIN	-		
SOD1	153	P00441	11	GDGPVQGIINFEQKE	-		
SOD1	153	P00441	16	QGIINFEQKESNGPV	-		
SOD1	153	P00441	21	FEQKESNGPVKVWGS	-		
SOD1	153	P00441	26	SNGPVKVWGSIKGLT	-		
SOD1	153	P00441	31	KVWGSIKGLTEGLHG	-		
SOD1	153	P00441	36	IKGLTEGLHGFHVHE	-		
SOD1	153	P00441	41	EGLHGFHVHEFGDNT	-		
SOD1	153	P00441	46	FHVHEFGDNTAGCTS	-		
SOD1	153	P00441	51	FGDNTAGCTSAGPHF	-		
SOD1	153	P00441	56	AGCTSAGPHFNPLSR	-		
SOD1	153	P00441	61	AGPHFNPLSRKHGGP	-		
SOD1	153	P00441	66	NPLSRKHGGPKDEER	-		
SOD1	153	P00441	71	KHGGPKDEERHVGDL	-		
SOD1	153	P00441	76	KDEERHVGDLGNVTA	-		
SOD1	153	P00441	81	HVGDLGNVTADKDGV	-		
SOD1	153	P00441	86	GNVTADKDGVADVSI	-		
SOD1	153	P00441	86	GNVTADKDGVADVXI	-	X=phosphoserine aa99	
SOD1	153	P00441	91	DKDGVADVSIEDSVI	-		
SOD1	153	P00441	91	DKDGVADVXIEDSVI	-	X=phosphoserine aa99	
SOD1	153	P00441	91	DKDGVADVSIEDXVI	-	X=phosphoserine aa103	
SOD1	153	P00441	96	ADVSIEDSVISLSGD	-		
SOD1	153	P00441	101	EDSVISLSGDHCIIG	-		
SOD1	153	P00441	101	EDSVIXLSGDHCIIG	-	X=phosphoserine aa106	
SOD1	153	P00441	101	EDSVISLXGDHCIIG	-	X=phosphoserine aa108	
SOD1	153	P00441	106	SLSGDHCIIGRTLVV	-		
SOD1	153	P00441	111	HCIIGRTLVVHEKAD	-		
SOD1	153	P00441	116	RTLVVHEKADDLGKG	-		
SOD1	153	P00441	121	HEKADDLGKGGNEES	-		
SOD1	153	P00441	126	DLGKGGNEESTKTGN	-		
SOD1	153	P00441	131	GNEESTKTGNAGSRL	-		
SOD1	153	P00441	136	TKTGNAGSRLACGVI	-		
SOD1	153	P00441	140	NAGSRLACGVIGIAQ	-		

LRRK2	2527	Q5S007	41	DLLVFTYSERASKLF	8.2		
LRRK2	2527	Q5S007	61	HVPLLIVLDSYMRVA	2.3		
LRRK2	2527	Q5S007	66	IVLDSYMRVASVQQV	9		
LRRK2	2527	Q5S007	116	QLILKMLTVHNASVN	10		
LRRK2	2527	Q5S007	136	LKTLDLLLTSGKITL	15		
LRRK2	2527	Q5S007	156	ESDIFMLIFDAMHSF	15		
LRRK2	2527	Q5S007	161	MLIFDAMHSFPANDE	19		
LRRK2	2527	Q5S007	196	LTEFVENKDYMILLS	15		
LRRK2	2527	Q5S007	201	ENKDYMILLSALTNF	17		
LRRK2	2527	Q5S007	221	IVLHVLHCLHSLAIP	20		
LRRK2	2527	Q5S007	246	NVRCYNIVVEAMKAF	18		
LRRK2	2527	Q5S007	251	NIVVEAMKAFPMSER	13		
LRRK2	2527	Q5S007	281	NFFNILVLNEVHEFV	11		
LRRK2	2527	Q5S007	401	VMLSMLMHSSSKEVF	12		
LRRK2	2527	Q5S007	431	NFRKILLSKGIHLNV	5.8		
LRRK2	2527	Q5S007	476	SLDIMAAVVPKILTV	17		
LRRK2	2527	Q5S007	501	QLEALRAILHFIVPG	17		
LRRK2	2527	Q5S007	536	QCFKNDIHKLVLAAL	14		
LRRK2	2527	Q5S007	541	DIHKLVLAALNRFIG	8.3		
LRRK2	2527	Q5S007	561	KCGLKVISSIVHFPD	9.8		
LRRK2	2527	Q5S007	606	GLSLIGYLITKKNVF	17		
LRRK2	2527	Q5S007	611	GYLITKKNVFIGTGH	18		
LRRK2	2527	Q5S007	626	LLAKILVSSLYRFKD	11		
LRRK2	2527	Q5S007	646	TKGFQTILAILKLSA	18		
LRRK2	2527	Q5S007	651	TILAILKLSASFSKL	5.9		
LRRK2	2527	Q5S007	656	LKLSASFSKLLVHHS	15		
LRRK2	2527	Q5S007	661	SFSKLLVHHSFDLVI	17		
LRRK2	2527	Q5S007	671	FDLVIFHQMSSNIME	7.5		
LRRK2	2527	Q5S007	781	KGDSQIISLLLRRLA	13		
LRRK2	2527	Q5S007	791	LRRLALDVANNSICL	13		
LRRK2	2527	Q5S007	831	KQTNIASTLARMVIR	17		
LRRK2	2527	Q5S007	841	RMVIRYQMKSAVEEG	13		
LRRK2	2527	Q5S007	901	SFLVKKKSNSISVGE	18		
LRRK2	2527	Q5S007	911	ISVGEFYRDAVLQRC	19		
LRRK2	2527	Q5S007	1041	DLHSNKFTSFPSYLL	16		
LRRK2	2527	Q5S007	1051	PSYLLKMSCIANLDV	19		
LRRK2	2527	Q5S007	1086	KQFNLSYNQLSFVPE	14		
LRRK2	2527	Q5S007	1126	LRLKELKILNLSKNH	20		
LRRK2	2527	Q5S007	1131	LKILNLSKNHISSLS	17		
LRRK2	2527	Q5S007	1156	ESFSARMNFLAAMPF	15		
LRRK2	2527	Q5S007	1161	RMNFLAAMPFLPPSM	11		
LRRK2	2527	Q5S007	1176	TILKLSQNKFSCIPE	19		
LRRK2	2527	Q5S007	1221	NLRELLFSHNQISIL	12		
LRRK2	2527	Q5S007	1241	AYLWSRVEKLHLSHN	15		
LRRK2	2527	Q5S007	1246	RVEKLHLSHNKLKEI	19		
LRRK2	2527	Q5S007	1316	KDIIRFLQQRLKKAV	17		
LRRK2	2527	Q5S007	1326	LKKAVPYNRMKLMIV	12		
LRRK2	2527	Q5S007	1396	AGREEFYSTHPHFMT	20		
LRRK2	2527	Q5S007	1406	PHFMTQRALYLAVYD	4		
LRRK2	2527	Q5S007	1501	RKTIINESLNFKIRD	15		
LRRK2	2527	Q5S007	1551	KRLLQLVRENQLQLD	13		
LRRK2	2527	Q5S007	1571	HAVHFLNESGVLLHF	13		
LRRK2	2527	Q5S007	1581	VLLHFQDPALQLSDL	19		
LRRK2	2527	Q5S007	1601	KWLCKIMAQILTVKV	18		
LRRK2	2527	Q5S007	1646	MSQYFKLLEKFQIAL	13		
LRRK2	2527	Q5S007	1661	PIGEEYLLVPSSLSD	17		
LRRK2	2527	Q5S007	1701	PMGFWSRLINRLLEI	11		
LRRK2	2527	Q5S007	1706	SRLINRLLEISPYML	18		
LRRK2	2527	Q5S007	1761	SFLKITVPSCRKGCI	19		
LRRK2	2527	Q5S007	1906	KIFNKHTSLRLLRQE	8.4		
LRRK2	2527	Q5S007	1921	LVVLCHLHHPSLISL	19		
LRRK2	2527	Q5S007	1931	SLISLLAAGIRPRML	14		
LRRK2	2527	Q5S007	1941	RPRMLVMELASKGSL	15		

LRRK2	2527	Q5S007	1971	QHRIALHVADGLRYL	16		
LRRK2	2527	Q5S007	1976	LHVADGLRYLHSAMI	12		
LRRK2	2527	Q5S007	1981	GLRYLHSAMIIYRDL	3.3		
LRRK2	2527	Q5S007	1986	HSAMIIYRDLKPHNV	18		
LRRK2	2527	Q5S007	1991	IYRDLKPHNVLLFTL	20		
LRRK2	2527	Q5S007	2001	LLFTLYPNAAIIAKI	8		
LRRK2	2527	Q5S007	2061	LLLYDILTTGGRIVE	17		
LRRK2	2527	Q5S007	2271	KLAIFEDKTVKLKGA	12		
LRRK2	2527	Q5S007	2276	EDKTVKLKGAAPLKI	12		
LRRK2	2527	Q5S007	2321	TKIFSFSNDFTIQKL	9.7		
LRRK2	2527	Q5S007	2331	TIQKLIETRTSQLFS	19		
LRRK2	2527	Q5S007	2351	DSNIITVVVDTALYI	18		
LRRK2	2527	Q5S007	2431	GGHILLLDLSTRRLI	5.5		
LRRK2	2527	Q5S007	2436	LLDLSTRRLIRVIYN	18		
LRRK2	2527	Q5S007	2441	TRRLIRVIYNFCNSV	20		
LRRK2	2527	Q5S007	2446	RVIYNFCNSVRVMMT	7.9		
LRRK2	2527	Q5S007	2451	FCNSVRVMMTAQLGS	20		
